# Operator-level quantum acceleration of non-logconcave sampling

**DOI:** 10.1073/pnas.2512789123

**Published:** 2026-02-20

**Authors:** Jiaqi Leng, Zhiyan Ding, Zherui Chen, Lin Lin

**Affiliations:** ^a^Simons Institute for the Theory of Computing, University of California, Berkeley, CA 94720; ^b^Department of Mathematics, University of California, Berkeley, CA 94720; ^c^Department of Mathematics, University of Michigan, Ann Arbor, MI 48109; ^d^Applied Mathematics and Computational Research Division, Lawrence Berkeley National Laboratory, Berkeley, CA 94720

**Keywords:** Gibbs sampling, Langevin dynamics, Witten Laplacian, singular value thresholding, quantum algorithms

## Abstract

Sampling from probability distributions of the form $\sigma \propto e^{-\beta V}$, where V is a continuous potential, is a fundamental task across physics, chemistry, biology, computer science, and statistics. However, when V is nonconvex, the resulting distribution becomes non-logconcave, and classical methods such as Langevin dynamics often exhibit poor performance. We introduce a quantum algorithm that provably accelerates a broad class of continuous-time sampling dynamics. For Langevin dynamics, our method encodes the target Gibbs measure into the amplitudes of a quantum state, identified as the kernel of a block matrix derived from a factorization of the Witten Laplacian operator. This connection enables Gibbs sampling via singular value thresholding and yields up to a quartic quantum speedup over best-known classical Langevin-based methods in the non-logconcave setting. Building on this framework, we further develop the first quantum algorithm that accelerates replica exchange Langevin diffusion, a widely used method for sampling from complex, rugged energy landscapes.

## Significance

Sampling from Gibbs distri-butions under continuous, often nonconvex, potentials is a fundamental challenge, which hinders classical methods such as the Langevin dynamics. Existing quantum approaches primarily rely on quantum walks to accelerate classical sampling algorithms, which limits the design space of quantum algorithms to the choice of classical counterparts and inherits technical difficulties in error analysis due to temporal discretization. We introduce a versatile framework for accelerating general sampling processes using quantum computers. In particular, our quantum algorithm is constructed at the operator level without relying on time discretization, thereby providing a path for quantizing continuous-time processes.

Given a continuous potential function $V:R^{d}\to R$ and an inverse temperature β, sampling from the Gibbs distribution $\sigma \propto e^{-\beta V}$ is an important task in computational sciences. Originally rooted in statistical physics, Gibbs sampling has found widespread applications in computational chemistry (1), statistics (2–4), and learning theory (5, 6). Langevin dynamics is a standard continuous-time process for Gibbs sampling. The overdamped Langevin equation is given by

[1] $$\begin{array}{c} \text{d}X_{t}=-\nabla V\left(X_{t}\right)\;\;\text{d}t+\sqrt{2/\beta }\;\;\text{d}W_{t}, \end{array}$$

where $W_{t}$ is the standard d-dimensional Brownian motion. The Gibbs measure σ is the stationary distribution of Eq. **1**. This equation can be approximately solved using a simple scheme known as the Euler–Maruyama discretization, or the unadjusted Langevin algorithm (ULA):

[2] $$\begin{array}{c} X_{n+1}=-\nabla V\left(X_{n}\right)\Delta t_{n}+\sqrt{2/\beta }\Delta W_{n}, \end{array}$$

where $\Delta t_{n}=t_{n+1}-t_{n}$ is a time step, and $\Delta W_{n}∼N\left(0,\Delta t_{n}I_{d}\right)$ follows Gaussian distribution. While ULA approximates the continuous dynamics Eq. **1**, its discretization can be unstable, which requires a careful choice of time steps to accurately approximate the target stationary distribution. Metropolis-Adjusted Langevin Algorithm (MALA), a variant of ULA, incorporates a Metropolis–Hastings correction step to ensure the correct equilibrium, yielding a high-accuracy, discrete-time Gibbs sampler (7, 8).

The performance of discretized dynamics (e.g., ULA, MALA, and variants) has been extensively studied (9–21). Although the exponential convergence of the continuous dynamics Eq. **1** to the Gibbs measure can be established under the sole assumption that σ satisfies a Poincaré inequality, analyzing these discrete-time Markov chain Monte Carlo (MCMC) processes is often much more technical and relies on stronger assumptions (22).

In applications like molecular simulation and training generative models, the potential V is often nonconvex, producing a non-logconcave distribution σ. Sampling such distributions is difficult for both continuous- and discrete-time Langevin dynamics: convergence can be exponentially slow in the barrier height of V and the inverse temperature β, a phenomenon known as metastability (10, 23, 24) and is closely related to rare event sampling (25–33). To overcome such challenges, advanced sampling algorithms such as replica exchange have been developed (34–41). They leverage more than one temperature level to facilitate transitions over barriers that are otherwise prohibitive at low temperatures. While nonasymptotic convergence results of replica-exchange-type algorithms exist for certain highly structured models [e.g., Gaussian mixture (42), mean-field spins (43), multimodal distributions (44, 45), etc.], a general understanding of theoretical efficiency guarantees remains largely open.

Quantum computers offer a promising alternative for accelerating classical sampling processes. The quantum walk algorithm, first developed by Szegedy (46) two decades ago, can be used to achieve a quadratic speedup over classical discrete-time MCMC processes in query complexity. To our knowledge, existing quantum algorithms for classical Gibbs sampling also apply the quantum walk to quantumly encode classical discrete-time MCMC processes, such as ULA, MALA, and their variants (47–49). This restricts the design space of quantum algorithms to classical MCMC frameworks. In addition, the query complexity analysis highly relies on the complexity of the classical discrete-time MCMC process, which inherits the challenges of analyzing the convergence of these discrete-time schemes.

In this work, we propose a framework for achieving quantum acceleration of general sampling processes with the Gibbs measure σ as a stationary distribution. Our quantum algorithm is constructed at the operator level *without* relying on the dynamics generated by a discrete-time classical MCMC process. Take the Langevin dynamics Eq. **1** for example. Our method starts from the Fokker–Planck equation (for general Markov processes, it is called the Kolmogorov forward equation):

[3] $$\begin{array}{c} \partial_{t}\rho =L\left(\rho \right):=\nabla \cdot \left(\rho \left(t,x\right)\nabla V\left(x\right)\right)+\beta^{-1}\Delta \rho \left(t,x\right). \end{array}$$

Here, ρ(t,x) represents the probability density associated with the random variable $X_{t}$, and the Gibbs measure is a stationary point of the dynamics, satisfying L(σ)=0. We refer the reader to *SI Appendix*, section A for the relevant mathematical preliminaries.

**Fig. 1.**
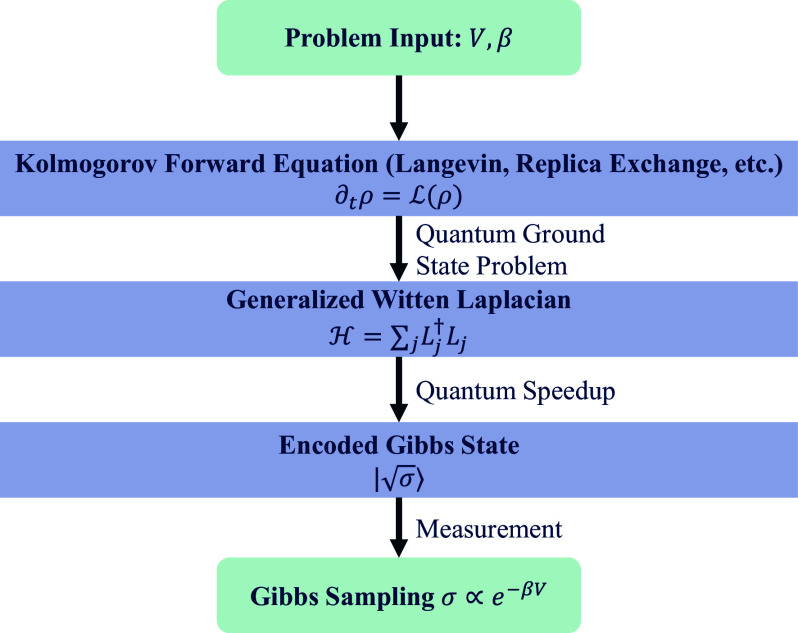
A schematic diagram of operator-level quantum acceleration of classical Gibbs sampling.

After a similarity transformation, the generator L can be mapped to a quantum Hamiltonian H, known as the Witten Laplacian operator (50), up to a constant scaling factor. The Gibbs measure is then encoded as the ground state of H, denoted by $|\sqrt{\sigma }\rangle$. Measuring this ground state in the computational basis yields |x〉 with a probability proportional to σ(x), thereby achieving Gibbs sampling (Fig. 1).

In order to efficiently prepare the ground state, a key structural property of H is that it is frustration-free, meaning that it can be decomposed as a sum of operators: $H=\sum_{i}L_{i}^{†}L_{i}$, and the encoded Gibbs state $|\sqrt{\sigma }\rangle$ is a simultaneous singular vector of all $L_{i}$’s, satisfying $L_{i}|\sqrt{\sigma }\rangle =0$. Each $L_{i}$ is a first-order differential operator free of time-discretization errors, and can be constructed directly from the gradient ∇V(x).

We will demonstrate that this frustration-free structure provides a simple mechanism to achieve quantum speedup with respect to the continuous-time Langevin dynamics in the non-logconcave setting, whose efficiency depends solely on the Poincaré constant (*Theorem* 1). Because of the broad applications of continuous sampling problems and the effectiveness of Langevin dynamics, extensive research has examined the complexity of Langevin-based sampling algorithms under various geometric assumptions on the target distribution-such as strong log-convexity (19, 47), log-Sobolev (48, 51), Cheeger (48, 51), and Poincaré typed inequalities (52)-in both quantum and classical settings. Table 1 compares the query complexities of our algorithms with existing quantum and classical algorithms. Although there is a vast body of literature on Langevin-based samplers, our algorithm is the first (classical or quantum) algorithm that provably achieves the $C_{\mathrm{PI}}^{1/2}$ scaling for a target distribution with a Poincaré constant $C_{\mathrm{PI}}$. In contrast to previous quantum algorithms (47, 48), our operator-level formulation bypasses time-discretization error analysis and enables a direct continuous analysis via the Witten Laplacian. As a result, we obtain complexity bounds expressed directly in terms of the Poincaré constant of the invariant measure, without relying on additional geometric or isoperimetric assumptions such as Cheeger-type inequalities. We do not expect the geometric-constant dependence appearing in refs. 47 and 48 to admit substantial improvement, since in the strongly convex regime one has the identity $\gamma^{-1}=C_{\mathrm{CG}}^{2}=C_{\mathrm{PI}}$. On the other hand, for a broad class of highly nonconvex potentials, $C_{\text{PI}}=\tilde{\Theta }\left(C_{\text{CG}}\right)$, where $C_{\mathrm{CG}}$ is the Cheeger constant (defined in *SI Appendix*, section B), implying that our quantum algorithm can achieve a quartic speedup over MALA for non-logconcave sampling tasks.^*^ Beyond Langevin-based sampling algorithms, the proximal method achieves a query complexity linear in $C_{\mathrm{PI}}$ by doubling the sampling space and employing a careful resampling process (52). Obtaining a comparable complexity analysis within the framework of Langevin dynamics, to the best of our knowledge, remains an open problem. A more detailed discussion and comparison can be found in *SI Appendix*,section B.

**Table 1.** Summary of query complexities of some quantum and classical algorithms for solving the sampling problem when β=1

Algorithms	Platform	Assumption	Complexity	Warm start
Ours (*Theorem* 1)	Quantum	$C_{\mathrm{PI}}$-Poincaré	$\tilde{O}\left(d^{1/2}C_{\mathrm{PI}}^{1/2}\right)$	Yes
Quantum MALA v1 (47)	Quantum	γ-strongly convex	$\tilde{O}\left(d^{1/4}/\gamma^{1/2}\right)$	Yes
Quantum MALA v2 (48)	Quantum	$C_{\mathrm{CG}}$-Cheeger	$\tilde{O}\left(d^{1/2}C_{\mathrm{CG}}\right)$	Yes
ULA (52)	Classical	$C_{\mathrm{PI}}$-Poincaré	$\tilde{O}\left(dC_{\mathrm{PI}}^{2}/\epsilon^{2}\right)$	No
Proximal (52)	Classical	$C_{\mathrm{PI}}$-Poincaré	$\tilde{O}\left(d^{1/2}C_{\mathrm{PI}}\right)$	No
MALA (19)	Classical	γ-strongly convex	$\tilde{O}\left(d^{1/2}/\gamma \right)$	Yes
MALA (51)	Classical	$C_{\mathrm{CG}}$-Cheeger	$\tilde{O}\left(dC_{\mathrm{CG}}^{2}\right)$	No

The precision parameter ϵ measures the difference (e.g., TV distance) between the target Gibbs measure and the output random variable. Here, $\tilde{O}$ hides the logarithmic dependence on the precision parameter ϵ. Our warm start assumption is consistent with ref. (48, Remark 27] and strictly weaker than that in ref. (47, (C.15)], as discussed in *SI Appendix*, Remark 20.

To ensure high success probability, our algorithm requires a good initial state, or a “warm start,” which has a constant $L^{2}$-overlap with the target state $|\sqrt{\sigma }\rangle$. This is a standard assumption in the quantum sampling literature (47, 48), and strictly weaker than common warm-start assumptions (e.g, $\chi^{2}$-divergence) in classical literature (19, 52). Several established strategies exist for preparing such states, including quantum simulated annealing (48, 53) and variational quantum circuits. In this work, we propose an approach to prepare this warm-start state based on Lindblad dynamics. Specifically, we observe that a Lindblad master equation employing the $L_{j}$ operators (i.e., factors of the Witten Laplacian) as jump operators naturally recovers the Fokker–Planck equation. For potential functions V with moderately simple energy landscapes (for example, those with a constant number of local minima), short-time evolution under this Lindblad dynamics suffices to prepare a warm-start state.

The framework described above can be extended to a broad class of stochastic processes whose infinitesimal generators admit a similar quantization via a similarity transformation, thereby providing a natural and efficient means to accelerate the continuous-time sampling methods without introducing time-discretization error. In such cases, the quantum Hamiltonian H is referred to as the generalized Witten Laplacian. As a concrete demonstration, we apply our method to accelerate the replica exchange Langevin diffusion (*Theorem* 2), which leads to the first provable quantum speedup of continuous-time enhanced sampling dynamics.

## Poincaré Constant.

The generator L of the Langevin dynamics Eq. **3** is detailed balanced, namely, the adjoint of the operator L, denoted as $L^{†}$, is self-adjoint with respect to an inner product related to the stationary distribution σ, defined as ${\left\langle f,g\right\rangle }_{\sigma }:=\int_{R^{d}}\left(fg\right)\sigma \;\text{d}x$. The smallest positive eigenvalue of $-L^{†}$ is called the spectral gap, denoted as $\text{Gap}\left(L^{†}\right)$. The inverse of the spectral gap, denoted as $C_{\mathrm{PI}}$, is the Poincaré constant. A small spectral gap (or a large Poincaré constant) is a strong indicator that the mixing process can be slow.

**Fig. 2.**
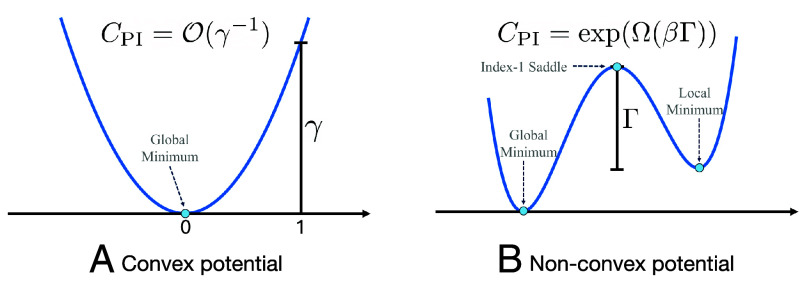
For Langevin dynamics, the Poincaré constant $C_{\mathrm{PI}}$ scales as $O(\gamma^{-1})$ for the convex potential, but as exp(Ω(βΓ)) for the nonconvex potential. The mixing time of the continuous-time Langevin dynamics scales linearly in $C_{\mathrm{PI}}$.

The spectral gap and the Poincaré constant have direct geometric interpretations. If the potential V(x) is γ-strongly convex (in this case, sampling from σ is referred to as logconcave sampling), the smallest eigenvalue for the Hessian of V is lower bounded by some constant γ>0, i.e., $\nabla^{2}V⪰\gamma I_{d}$, then $C_{\mathrm{PI}}=O\left(\gamma^{-1}\right)$ (54, Chapter 9] (55, Theorem 4.8.5]. Thus, the Poincaré constant decreases as the potential becomes more convex, causing the Gibbs distribution to concentrate more sharply around the global minimum (Fig. 2*A*).

## Operator-Level Acceleration of Langevin Dynamics

### Witten Laplacian.

From the generator L in Eq. **3**, we define a new operator ($\sigma^{\pm 1/2}$ act as multiplicative operators):

[4] $$\begin{array}{c} H=-\sigma^{-1/2}\;○\;L\;○\;\sigma^{1/2}=-\frac{1}{\beta }\Delta +\left(\frac{{\beta \|\nabla V\|}^{2}}{4}-\frac{1}{2}\Delta V\right). \end{array}$$

Since $L^{†}$ is self-adjoint with respect to the σ-weighted inner product, H is self-adjoint with respect to the standard $L^{2}$ inner product. The operator H is called the Witten Laplacian,^†^ which was first proposed by Witten in his analytical proof of the Morse inequalities (50). Later, Witten Laplacian became a fundamental tool in the study of metastability (27, 56). We may readily check that when $V\left(x\right)=\gamma x^{2}/2$, the Witten Laplacian H is the Hamiltonian of a quantum harmonic oscillator with a gap γ, which is independent of β.

Due to the similarity transformation, the spectrum of H is the same as that of −L (and thus of $-L^{†}$), which belongs to the nonnegative real axis. The kernel of H is given by the encoded Gibbs state $|\sqrt{\sigma }\rangle$ with $\langle x|\sqrt{\sigma }\rangle =\sqrt{\sigma (x)}$, which is a normalized state with respect to the standard $L^{2}$ inner product. In other words, if the Langevin dynamics is ergodic, then $|\sqrt{\sigma }\rangle$ is the unique ground state of H with a spectral gap $\text{Gap}\left(H\right)=\;\text{Gap}\left(L^{†}\right)$. From the encoded Gibbs state we can readily evaluate any classical observable O(x) according to $\langle |\sqrt{\sigma }\rangle O|\sqrt{\sigma }\rangle =\int O\sigma \;\text{d}x$. Here, we extend the standard $L^{2}$ inner product to complex numbers and use the bra-ket notation $\langle f|g\rangle :=\langle f,g\rangle =\int \overset{¯}{f}g\;\;\text{d}x$, where $\overset{¯}{f}$ is the complex conjugation of f, and f,g can be interpreted as unnormalized quantum states.

From a PDE perspective, the operators L and H are related as follows:

[5] $$\begin{array}{cc} \partial_{t}\rho & =L\rho \;\Leftrightarrow \;\partial_{t}u=-H\left(u\right)\\ H(u)\;:= & \beta^{-1}\Delta u-\left(\frac{{\beta \|\nabla V\|}^{2}}{4}-\frac{1}{2}\Delta V\right)u,\;\rho =\sqrt{\sigma }u. \end{array}$$

Since both operators have the same spectral gap, corresponding to *SI Appendix*, Eq. S25, we have

[6] $$\begin{array}{c} \begin{array}{c} \chi^{2}\left(\rho \left(t\right),\sigma \right)={\left|u\left(t\right)-\sqrt{\sigma }\right|}^{2}=e^{-2\;\text{Gap}\left(H\right)t}\chi^{2}\left(\rho \left(0\right),\sigma \right). \end{array} \end{array}$$

Here, we use the fact that ${\left|u\left(t\right)-|\sqrt{\sigma }\rangle \right|}^{2}$ is equal to the $\chi^{2}$-divergence. Therefore, u(t) converges exponentially to the encoded Gibbs state in $\chi^{2}$-divergence.

### Quantum Algorithm.

The mapping from the generator of Langevin dynamics L to the Witten Laplacian H allows us to tackle the Gibbs sampling problem as a ground state preparation problem, which is a standard task in quantum computing (57–60). However, such algorithms inevitably require querying the potential term, $\frac{{\beta \|\nabla V\|}^{2}}{4}-\frac{1}{2}\Delta V$ in Eq. **5**, which depends on both the first- and second-order derivatives of the potential V. This is undesirable since the classical Langevin dynamics only require first-order information.

The Witten Laplacian admits the following factorization (50) (for j∈[d]={1,2,⋯,d}):

[7] $$\begin{array}{c} H=\sum_{j=1}^{d}L_{j}^{†}L_{j},\;L_{j}:=-i\frac{1}{\sqrt{\beta }}\partial_{x_{j}}-i\frac{\sqrt{\beta }}{2}\partial_{x_{j}}V.\; \end{array}$$

The encoded Gibbs state is simultaneously annihilated by all $L_{j}$’s, i.e., $L_{j}|\sqrt{\sigma }\rangle =0$. In other words, $|\sqrt{\sigma }\rangle$ is the ground state of each individual Hamiltonian term $L_{i}^{†}L_{i}$. Such a Hamiltonian H is called frustration-free.^‡^ By concatenating all operators $L_{i}$ together as

[8] $$\begin{array}{c} L:={\left[L_{1}^{⊤},L_{2}^{⊤},\cdots ,L_{d}^{⊤}\right]}^{⊤}, \end{array}$$

Eq. **7** can be compactly written as $H=L^{†}L$. Furthermore, constructing L only requires the first-order information of the potential V.

Based on this observation, we propose a quantum algorithm for the Gibbs sampling task without solving either the Fokker–Planck equation or the dynamics Eq. **5**. The main idea is to prepare the encoded Gibbs state $|\sqrt{\sigma }\rangle$ as the unique right singular vector of L associated with the zero singular value. The singular value gap of L is equal to $\text{Gap}\left(L\right)=\sqrt{\text{Gap}\left(H\right)}=1/\sqrt{C_{\mathrm{PI}}}$. This provides a source for quantum speedup at the operator level, without either spatial or temporal discretization.

To implement this algorithm on the quantum computer, quantities such as $|\sqrt{\sigma }\rangle$ and L must be spatially discretized (*SI Appendix*, section D.2). With some abuse of notation, the spatially discretized quantities are still denoted by the same symbols. The spatial discretization error depends on the smoothness property of V, which can be systematically controlled (*SI Appendix*, section D.1), and the discussion is omitted here for simplicity. We emphasize that, unlike classical MCMC processes, our algorithm does not require temporal discretization, which often presents significant challenges in the analysis.

Below, we briefly describe the major steps of our quantum algorithm.

1. Prepare an initial state |ϕ〉 satisfying the warm start condition, i.e., $\left|\langle \phi |\sqrt{\sigma }\rangle \right|=\text{Ω}(1)$.
2. Block-encode the (spatially discretized) matrix L using a quantum circuit.
3. Apply a quantum singular value thresholding (SVT) algorithm to |ϕ〉 to filter out contributions in |ϕ〉 corresponding to nonzero singular values of L. This succeeds in preparing a state $|g\rangle \approx |\sqrt{\sigma }\rangle$ with a constant success probability.
4. Measure the resulting state in the computational basis^§^ to obtain a sample x that approximately follows the Gibbs distribution σ.

**Fig. 3.**
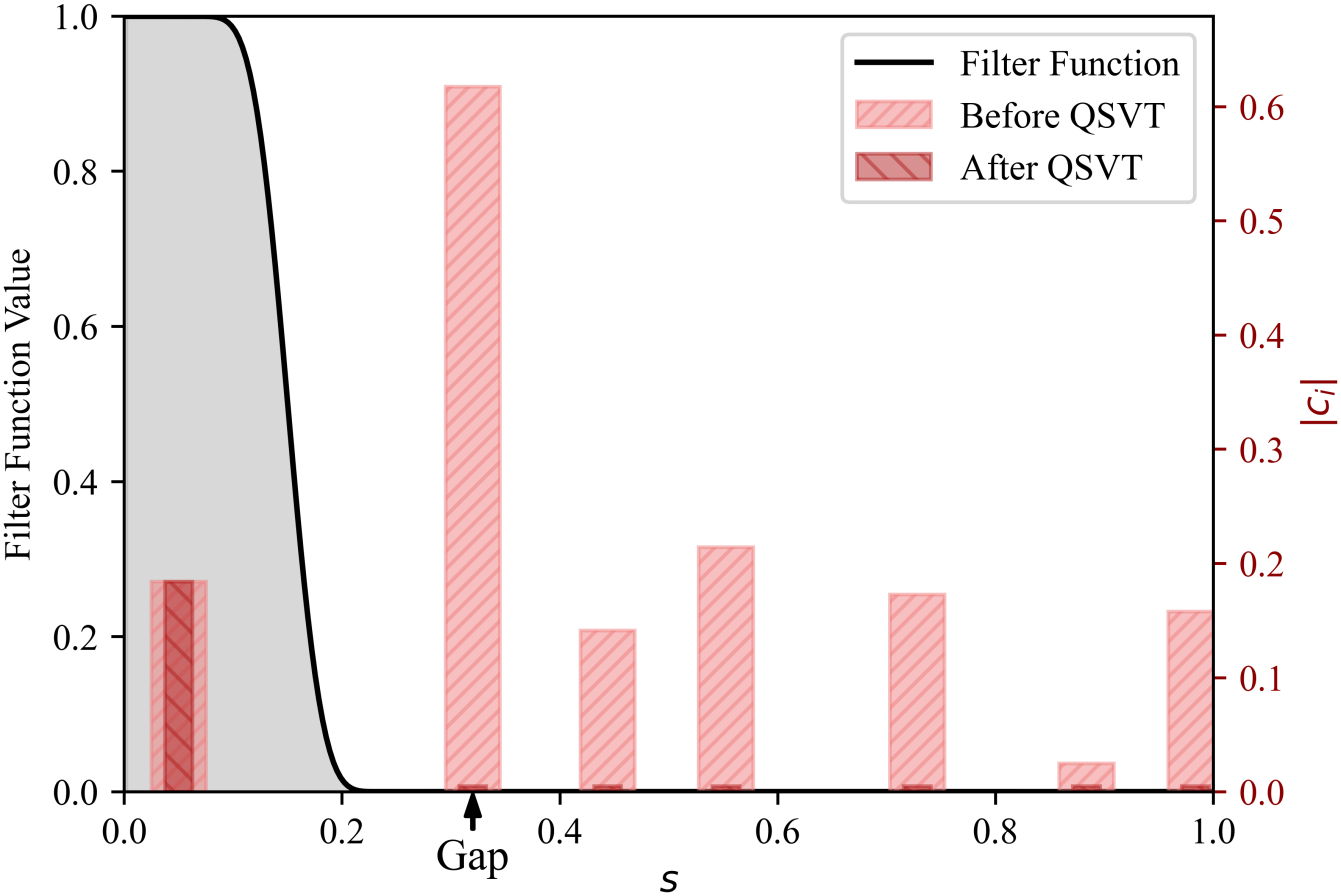
*Left* y-axis: Value of the filter function p(s) that is approximately equal to 1 on [0,Gap/4], and approximately equal to 0 on [3Gap/4,1]. *Right* y-axis: Projection $|c_{i}|$ to each right singular vector before and after QSVT. Here, $\text{Gap}=\;\text{Gap}\left(L\right)/\alpha$, and α is the block encoding subnormalization factor for L.

We now discuss the singular value thresholding algorithm in more detail. The spatially discretized matrix L is represented by its singular value decomposition (SVD) as $L=W\Sigma V^{†}$, where V is a unitary matrix, Σ is a diagonal matrix with nonnegative diagonal entries, and W is a rectangular matrix of orthogonal columns. This immediately gives the eigenvalue decomposition of $H=V\Sigma^{2}V^{†}$, i.e., the right singular vectors of L are the eigenvectors of H, and we identify $|\sqrt{\sigma }\rangle$ with the first column of V corresponding to the unique zero singular value. With the warm start assumption, we can write |ϕ〉=Vc with $|c_{1}|=\Omega \left(1\right)$. Then, we can leverage the Quantum Singular Value Transformation (QSVT) algorithm (61) to implement singular value thresholding. Suppose we have access to a block-encoding of L with a normalization factor α. We can construct an *even* polynomial p(s) that is approximately equal to 1 for $0\leq s\leq \;\text{Gap}\left(L\right)/4\alpha$, and approximately equal to 0 for $3\text{Gap}\left(L\right)/4\alpha \leq s\leq 1$, see Fig. 3. Applying QSVT with this even polynomial p(s) to the block-encoded L filters out all singular vectors with singular values greater than $3\;\text{Gap}\left(L\right)/4\alpha$, thereby leaving only the encoded Gibbs state. The details of the quantum algorithm, including background on the quantum singular value thresholding algorithm, are provided in *SI Appendix*, section C.

### Complexity Analysis.

Our quantum algorithm leverages two main subroutines: constructing the block-encoding of L and implementing singular value thresholding. Each $L_{j}$ can be represented as a pseudodifferential operator and discretized into a finite-dimensional matrix. We first truncate the space $R^{d}$ into a finite-sized domain $\Omega ={\left[-a,a\right]}^{d}$ and discretize it using a uniform grid of N points per dimension. Therefore, the dimension of each discretized $L_{j}$ operator is $N^{d}$. For a smooth potential V with certain growth conditions (e.g., $\left|V\left(x\right)\right|\geq {\gamma \|x\|}^{2}$ for some large x with γ>0), to achieve a target sampling accuracy ϵ in the TV distance, we can choose a=O(log(d/ϵ)) and N=a·polylog(1/ϵ). Since the value of the Gibbs measure is very close to zero on the boundary of Ω, we assume a periodic boundary condition to facilitate an efficient block-encoding of $L_{j}$ using Quantum Fourier Transforms, as detailed in *SI Appendix*, section D.1. It turns out that the discretized L can be block-encoded using 2 quantum queries to the gradient ∇V and 3 ancilla qubits, see *SI Appendix*, Proposition 17. For β≥1, the normalization factor of the block-encoding of L is equal to $\alpha =\tilde{O}\left(\sqrt{\beta d}\right)$, where $\tilde{O}\left(\cdot \right)$ suppresses poly-logarithmic factors in d and ϵ. Finally, we employ QSVT to implement singular value thresholding, which can be executed using $\tilde{O}\left(\alpha /\text{Gap}\left(L\right)\right)=\tilde{O}\left(\sqrt{\beta dC_{\mathrm{PI}}}\right)$ queries to the block-encoded matrix L. The overall complexity of our quantum algorithm is summarized as follows:

Theorem 1 (Informal). *Assuming access to a warm start state* |ϕ〉, *for a sufficiently large* β, *there exists a quantum algorithm that outputs a random variable distributed according to* η *such that* TV(η,σ)≤ϵ *using* $\sqrt{\beta dC_{\mathrm{PI}}}\cdot \text{poly}\log\left(d,1/\epsilon \right)$ *quantum queries to the gradient* ∇V.

A rigorous statement and proof of the theorem above are provided in *SI Appendix*, Theorem 18. To the best of our knowledge, this is the first algorithm that provably achieves a query complexity of $\tilde{O}\left(\sqrt{C_{\mathrm{PI}}}\right)$ for general potentials. In the presence of metastability, $C_{\mathrm{PI}}$ will grow exponentially with βΓ, where Γ denotes the barrier height of the potential V(x). This implies that our quantum algorithm can be used to e.g., simulate systems at a lower temperature.

## Operator-Level Acceleration of Replica Exchange

In this section, we extend the operator-level quantum algorithms to accelerate the replica exchange Langevin diffusion (RELD).

### Replica Exchange Langevin Diffusion.

For rugged (nonconvex) energy landscapes, Langevin dynamics may require prohibitively long times to traverse energy barriers separating local minima. Replica exchange (or parallel tempering) is a widely adopted strategy to mitigate this difficulty. It involves simulating multiple replicas at different temperatures, where high-temperature replicas explore the global landscape and low-temperature replicas concentrate near local minima. Through carefully designed swap moves between replicas, replica exchange enables barrier-crossing events that are otherwise rare at low temperatures. Its ability to accelerate overdamped Langevin diffusion is well established numerically (40, 41, 62, 63) and supported to a lesser extent by theoretical results, for instance in the setting of Gaussian mixtures (64).

For clarity, we focus on the case of replica exchange with two temperatures. The continuous-time formulation, called the replica exchange Langevin diffusion (RELD), can be modeled by two correlated Langevin dynamics with inverse temperatures $\beta >\beta^{\prime }>0$, respectively:

[9a] $$\begin{array}{c} \text{d}X_{t}=-\nabla V\left(X_{t}\right)\;\text{d}t+\sqrt{2/\beta }\;\text{d}W_{t}, \end{array}$$

[9b] $$\begin{array}{c} \text{d}Y_{t}=-\nabla V\left(Y_{t}\right)\;\text{d}t+\sqrt{2/\beta^{\prime }}\;\text{d}W_{t}^{\prime }, \end{array}$$

where $X_{t}$ and $Y_{t}$ denote the positions of the particle undergoing the low- and high-temperature Langevin dynamics, respectively. $W_{t}$ and $W_{t}^{\prime }$ represent two independent standard Brownian motions. The invariant distribution of the variable ${\left\{Z_{t}=\left(X_{t},Y_{t}\right)\right\}}_{t\geq 0}$ is a joint Gibbs measure:

[10] $$\begin{array}{c} \sigma \left(x,y\right)\propto \;\exp\left(-\beta V\left(x\right)-\beta^{\prime }V\left(y\right)\right). \end{array}$$

The marginal distribution of x under σ recovers the target distribution $\exp\left(-\beta V\left(x\right)\right)/Z_{\beta }$.

Without correlation, the mixing time of Eq. **9** is governed by the low-temperature (β) system, resulting in no acceleration. In RELD, an additional swap mechanism is introduced to accelerate convergence of Eq. **9** toward equilibrium. These swap events occur according to a Poisson clock and enable configuration exchange between replicas at different temperatures. Let μ>0 denote a swapping intensity, and we assume the sequential swapping events take place according to an exponential clock with a rate μ. At a swapping event time, the two particles swap their positions with a Metropolis-Hasting type probability:

[11] $$\begin{array}{c} s\left(X\left(t\right),Y\left(t\right)\right)=\;\min\left(1,\frac{\sigma (y,x)}{\sigma (x,y)}\right). \end{array}$$

Note that the Metropolis-Hasting type swapping does not change the invariant Gibbs measure σ.

To obtain a continuous-time dynamics that includes the exchange mechanism, let ρ(t,x,y) denote the probability density of the random variables $\left(X_{t},Y_{t}\right)$ for time t. The evolution of ρ(t,x,y) can be characterized by the forward Kolmogorov equation $\partial_{t}\rho =L\left(\rho \right)$, where the generator L captures both the Langevin dynamics and the swapping mechanism. In particular, L takes the following form:

[12] $$\begin{array}{c} L=L_{1}+L_{2}+L_{s}, \end{array}$$

where $L_{1}$ and $L_{2}$ are the Fokker–Planck generators in Eq. **3** with inverse temperatures β and $\beta^{\prime }$, respectively. $L_{s}$ corresponds to the Metropolis-Hasting swapping described by Eq. **12** with intensity μ as

[13] $$\begin{array}{c} L_{s}\left(\rho \left(x,y\right)\right):=\mu \left[s(y,x)\rho (y,x)-s(x,y)\rho (x,y)\right]. \end{array}$$

Under mild conditions, the dynamics can be shown to be ergodic, and the joint Gibbs measure σ is the unique invariant measure of the generator L (65).

For non-logconcave sampling, low-temperature Langevin dynamics often become trapped near metastable configurations for an exponentially long time. Through the swapping process, well-explored configurations in the high-temperature system can be transferred to the low-temperature system, thereby significantly reducing the overall mixing time (see ref. 42 for an analysis of RELD for Gaussian mixture models).

While the swapping mechanism enables replica exchange to effectively overcome energy barriers in the potential landscape and thus achieve faster mixing, it could still suffer from the curse of dimensionality. For example, refs. 44 and 45 suggest that in the presence of multiple modes, the spectral gap of replica exchange can decay rapidly as the problem dimension increases. Thus it is desirable to design a quantum algorithm with provable speedup over RELD.

### Generalized Witten Laplacian of RELD.

Now, we consider the operator transformation

[14] $$\begin{array}{c} H-\sigma^{-1/2}\;○\;L\;○\;\sigma^{1/2}, \end{array}$$

which is referred to as the generalized Witten Laplacian of RELD. Due to the similarity transformation, the spectrum of H is the same as that of −L, and the kernel of H is given by the extended encoded Gibbs state $|\sqrt{\sigma }\rangle$ with $\langle x,y|\sqrt{\sigma }\rangle =\sqrt{\sigma (x,y)}$. Note that $|\sqrt{\sigma }\rangle$ is a product state, and the Gibbs distribution $\sigma \propto e^{-\beta V}$ can be recovered by measuring $|\sqrt{\sigma }\rangle$ in the x-variable register. Under this transformation, the two Fokker–Planck-type operators $L_{1}$ and $L_{2}$ are mapped to two Witten Laplacians, denoted by $H_{1}$ and $H_{2}$. Similar to Eq. **7**, each Witten Laplacian can be factorized as the sum of d nonnegative operators of the form $L^{†}L$.

The transformation of the swap operator $L_{s}$ is more involved. First, we rewrite the operator $L_{s}=\mu \left(W-I\right)○S$, where I is the identity operator, W(ψ(x,y)):=ψ(y,x) interchanges the x and y variables in a function, and S(ψ(x,y)):=s(x,y)ψ(x,y) represents the multiplication with the function s. It can be readily verified that $\sqrt{s(x,y)\sigma (x,y)}=\sqrt{s(y,x)\sigma (y,x)}$. In other words, W commutes with $S^{1/2}\sigma^{1/2}$, i.e., $WS^{1/2}\sigma^{1/2}=S^{1/2}\sigma^{1/2}W$. Therefore, we can rewrite the transformed swap operator

[15] $$\begin{array}{cc} H_{s} & :=-\sigma^{-1/2}\;○\;L_{s}\;○\;\sigma^{1/2}=\mu S^{1/2}\left(I-W\right)S^{1/2}=L_{s}^{†}L_{s},\\ L_{s} & =\sqrt{\mu /2}\left(I-W\right)S^{1/2}. \end{array}$$

Note that we use $W^{2}=I$ in the last step. It turns out that the generalized Witten Laplacian of RELD consists of three component operators $H=H_{1}+H_{2}+H_{s}$. Moreover, H admits a factorization of the form $H=L_{\text{RE}}^{†}L_{\text{RE}}$, where

[16] $$\begin{array}{c} L_{\text{RE}}={\left[L_{1}^{⊤},\cdots ,L_{d}^{⊤},{\left(L_{1}^{\prime }\right)}^{⊤},\cdots ,{\left(L_{d}^{\prime }\right)}^{⊤},L_{s}^{⊤}\right]}^{⊤}, \end{array}$$

where the block matrix $L_{\text{RE}}$ encompasses (2d+1) operators: ${\left\{L_{j}\right\}}_{j=1}^{d}$ and ${\left\{L_{j}^{\prime }\right\}}_{j=1}^{d}$ correspond to the Langevin dynamics with inverse temperature β and $\beta^{\prime }$, respectively; $L_{s}$ corresponds to the swap mechanism. More details of $L_{\text{RE}}$ can be found in *SI Appendix*, section E.1. We refer to H as the generalized Witten Laplacian of RELD, and it enables us to apply our operator-level quantum sampling algorithm.

### Quantum Algorithms and Complexity Analysis.

The encoded (joint) Gibbs state $|\sqrt{\sigma }\rangle$ can be prepared as the ground state of the generalized Witten Laplacian H Eq. **17**, or equivalently, the right singular vector of $L_{\mathrm{RE}}$ associated with the zero singular value. This can be realized via the singular value thresholding algorithm as discussed before.

To construct a block-encoding of $L_{\mathrm{RE}}$, we need to perform spatial discretization for the component operators $L_{j}$ in $L_{\mathrm{RE}}$ for each j∈[2d+1]. Note that the component operator $L_{j}$ in $L_{\mathrm{RE}}$ acts on functions in $R^{2d}$. Similar to the previous section, we can first truncate the space $R^{2d}$ into a finite-size numerical domain $\Omega ={\left[-a,a\right]}^{2d}$ and discretize it using a uniform grid with $N^{2d}$ points. The discretized $L_{j}$ operator is a matrix of dimension $N^{2d}$. For a smooth potential with certain growth conditions, to achieve a target sampling accuracy ϵ in the TV distance, we can choose a=O(log(d/ϵ)) and N=a·polylog(1/ϵ).

Since the first 2d component operators in $L_{\mathrm{RE}}$ are essentially the same as in the Langevin dynamics case, they can be block-encoded using 2 queries to ∇V through the same technique as mentioned before. The last operator $L_{s}\propto \left(I-W\right)\;○\;S^{1/2}$ can be implemented by concatenating two quantum circuits, one for I−W and another for the scalar multiplication $S^{1/2}$. The exchange operator W can be realized by $d\lceil \log_{2}\left(N\right)\rceil$ SWAP gates; the scalar multiplication operator $S^{1/2}$ can be directly implemented using an arithmetic circuit and queries to the function V. Overall, we can build a block-encoding of $L_{\text{RE}}$ with 2 quantum queries to the gradient ∇V, 4 quantum queries to the function value of V, and 10 ancilla qubits, as detailed in *SI Appendix*, Proposition 21. The normalization factor of this block-encoding is equal to $\alpha \leq \tilde{O}\left(\sqrt{\beta d}\right)$ (suppressing poly-logarithmic factors in d and ϵ). Finally, we can implement QSVT for singular value thresholding, which creates a projection onto the encoded Gibbs state $|\sqrt{\sigma }\rangle$ using $\tilde{O}\left(\sqrt{\beta d/\;\text{Gap}\left(L^{†}\right)}\right)$ queries to the block-encoded matrix $L_{\mathrm{RE}}$. Here, $\text{Gap}\left(L^{†}\right)$ denotes the spectral gap of the generator of RELD, i.e., the smallest positive eigenvalue of $-L^{†}$. The overall complexity of the quantum algorithm is summarized below:

Theorem 2 (Informal). *Assuming access to a warm start state* ϕ, *and let* $\beta^{\prime }$, μ *be constant independent of* d *and* β. *For a sufficiently large* β, *there exists a quantum algorithm that outputs a random variable* $\left(X,Y\right)\in R^{2d}$ *distributed according to* η *such that* TV(η,σ)≤ϵ *using* $\sqrt{\beta d/\;\text{Gap}\left(L^{†}\right)}\cdot \text{poly}\log\left(d,1/\epsilon \right)$ *quantum queries to the function value* V *and the gradient* ∇V, *respectively. Consequently, the distribution of* X *with a marginal law* η *satisfies* TV(η,σ)≤ϵ, *where* $\sigma \propto e^{-\beta V}$.

A rigorous statement and proof of the theorem above are provided in *SI Appendix*, Theorem 23. The complexity in the above theorem scales as $O\left(\sqrt{1/\;\text{Gap}}\right)$, where Gap denotes the spectral gap of the continuous-time RELD.

## Lindblad Dynamics for Warm-Start Preparation

We consider a Lindblad master equation given by:

[17] $$\begin{array}{c} \partial_{t}\rho =L\left[\rho \right],\;L\left[\rho \right]\sum_{j=1}^{d}\left(2L_{j}\rho L_{j}^{†}-\left\{L_{j}^{†}L_{j},\rho \right\}\right), \end{array}$$

where ρ(t) is a time-dependent density operator and the jump operators ${\left\{L_{j}\right\}}_{j=1}^{d}$ are the same as in Eq. **7**. Here, {A,B}=AB+BA represents the anticommutator. It is straightforward to verify that the encoded Gibbs state is a fixed point of the Lindbladian L, since $L_{j}|\sqrt{\sigma }\rangle =0$ for all j. In general, ρ(t) is a mixed state. However, the evolution of its “diagonal elements” in the computational basis reduces to the Fokker–Planck equation in Eq. **3**; see *SI Appendix*, Lemma 24.

Although the Lindblad dynamics is not asymptotically faster in convergence, it can rapidly prepare a quantum representation of a metastable state. This behavior arises because, while the spectral gaps between metastable states are typically small, which leads to a large Poincaré constant and hence slow overall convergence, the metastable subspace itself is often well separated from the rest of the Lindbladian spectrum. This can happen for potential functions V with a relatively small number of local minima and for temperatures that are not too low. As a result, the system quickly relaxes into this low-lying subspace, yielding an intermediate state with a sufficiently large overlap with the target Gibbs state, which serves as an effective warm start for the subsequent quantum acceleration stage.

Therefore, by simulating the Lindblad equation on a quantum computer, which uses the same block-encoding of L (66), we can efficiently obtain a warm-start state for our SVT-based quantum algorithm. A numerical demonstration of this two-phase behavior is presented in the next section, and further technical details are provided in *SI Appendix*, section F.

## Numerical Experiments

In this section, we numerically demonstrate our quantum algorithms to accelerate Langevin dynamics (LD) and replica exchange Langevin diffusion (RELD) for non-logconcave sampling. Our results show that the quantum algorithms exhibit significant speedups over their classical counterparts. To simulate our quantum algorithms, we numerically implement singular value thresholding. The details of the numerical implementation can be found in *SI Appendix*, section G. Code and data supporting this study are available at (67)

### Quantum-Accelerated Langevin Dynamics.

We compare the performance of our quantum-accelerated Langevin sampling with that of MALA using the Müller-Brown potential (68), which is often used as a proof of concept for rare event sampling in computational chemistry. The Müller-Brown potential is characterized by a highly nonconvex energy surface with three local minima. Details of this potential function, including its analytical expression and energy landscape, are provided in *SI Appendix*, section G.2.

To conduct a fair comparison, both algorithms start from the same Gaussian distribution $\rho_{0}$ (or the corresponding Gaussian state $|\sqrt{\rho_{0}}\rangle$) centered at one of the local minima of the potential function. We choose three inverse temperatures: β=0.4,0.6,0.8 for the experiment. For all tested inverse temperatures, the overlap between the initial distribution $\rho_{0}$ and the target Gibbs state σ, i.e., $\left|\langle \sqrt{\rho_{0}}|\sqrt{\sigma }\rangle \right|$, is approximately 0.1, indicating a reasonable warm start.

**Fig. 4.**
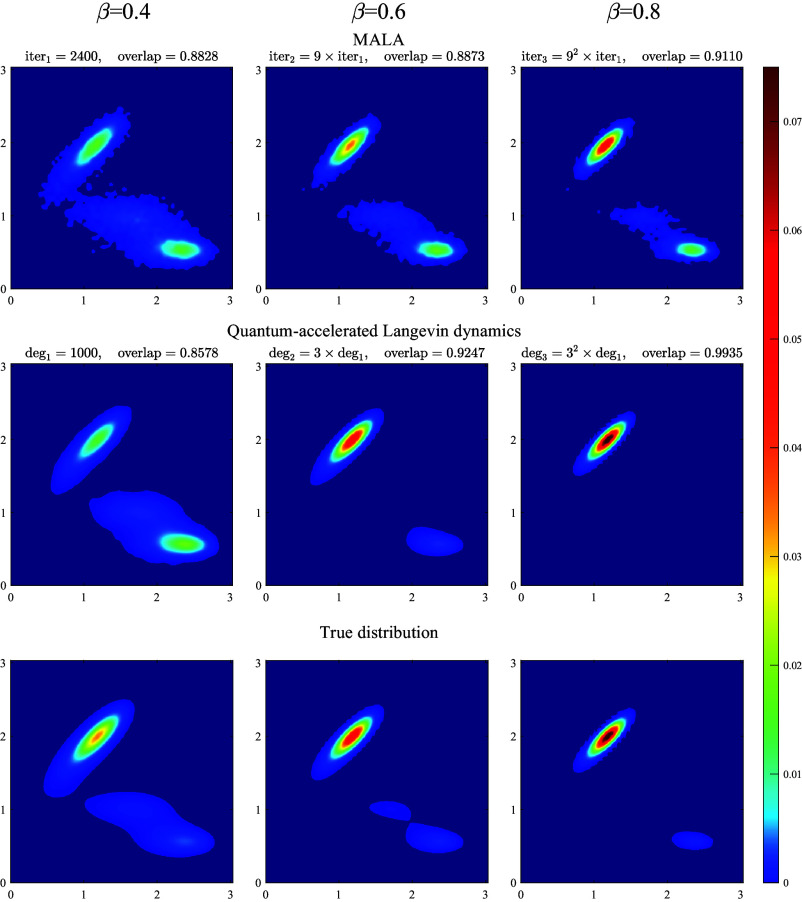
Quantum acceleration of Langevin dynamics. Each column corresponds to a different inverse temperature β. The overlap $\left|\langle \sqrt{\sigma }||\phi \rangle \right|$ measures the similarity between the sampled and true distribution, where $\langle \sqrt{\sigma }|$ is the encoded Gibbs state and |ϕ〉 represents either 1) the square root of the output probability density by MALA, or 2) the output pure state by our method. Note that the overlap definition can be generalized to accommodate mixed states; see Eq. **19**. *Top* row: Sample distributions obtained using MALA with the number of iterations increasing by a factor of 9. *Middle* row: Distributions obtained by our quantum algorithm with the degree of polynomials increasing by a factor of 3. *Bottom* row: True distribution (Gibbs state).

To reflect the computational complexity of the algorithms, we report the number of MALA iterations and the polynomial degree of the filter function in QSVT, both of which directly determine the number of queries to ∇V. Due to the nonconvexity of the potential, the Arrhenius law predicts that the mixing time of MALA scales exponentially with the inverse temperature. For an ascending sequence β∈{0.4,0.6,0.8}, the numerical results show that MALA requires approximately 9 times more iterations for every 0.2 increase in β, as illustrated in the *Top* row of Fig. 4. On the other hand, the *Middle* row of Fig. 4 shows that our quantum algorithm outputs a sample distribution with a comparable (or even slightly better) accuracy by increasing the QSVT polynomial degrees by a factor of 3 for the same increment in β. This observation highlights the efficiency of our quantum algorithm and demonstrates the desired speedup in query complexity over classical MALA.

### Quantum-Accelerated Replica Exchange.

**Fig. 5.**
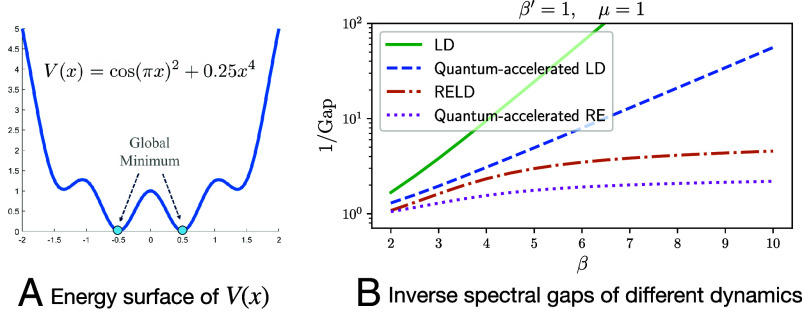
Quantum acceleration of replica exchange. *Left*: a nonconvex 1D potential. *Right*: Inverse spectral gaps for LD, RELD, and their quantum counterparts as functions of β. The spectral gap of LD decays exponentially in β, while that of RELD shows a much milder decay. The quantum algorithms achieve square-root improvements in the gaps in both cases.

We numerically demonstrate the quantum-accelerated RELD using a 1-dimensional potential:

[18] $$\begin{array}{c} V\left(x\right)=\;\cos{\left(\pi x\right)}^{2}+0.25x^{4}, \end{array}$$

which is a nonconvex function with 4 local minima, as illustrated in Fig. 5*A*. Even though this experiment is conducted on a one-dimensional example, we expect that similar behavior can be observed in a high dimensional setting, when the energy barrier can be identified along a “reaction coordinate” as is often the case in computational chemistry (69, 70).

In Fig. 5*B*, we illustrate the inverse spectral gaps of two classical dynamics (LD and RELD) and their quantum counterparts. The inverse spectral gap indicates the scaling of the query complexity (for both quantum and classical algorithms) as β grows. The spectral gaps of LD and RELD are computed based on the Witten Laplacians, i.e., Eqs. **4** and **15**, respectively. For the two corresponding quantum algorithms, the singular value gaps are computed using Eqs. **8** and **17**, respectively. For RELD, we set $\beta^{\prime }=\mu =1$ for all choices of β.

We observe that the spectral gap of Langevin dynamics (LD) decays exponentially as β increases, while RELD exhibits a much milder decay, illustrating the advantage of RELD for non-logconcave sampling. Furthermore, we find that the singular value gaps of the quantum-accelerated algorithms are approximately^¶^ the square root of the spectral gaps of their classical counterparts, consistent with our theoretical analysis and confirming that our quantum algorithms achieve a quadratic speedup as β becomes large.

**Fig. 6.**
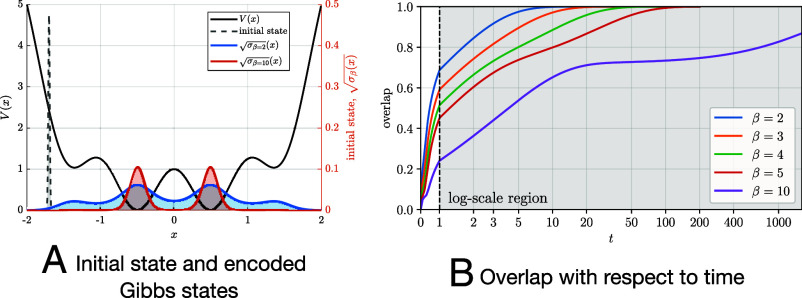
Lindbladian-based warm-start generation. *Left*: Initial state for the Lindblad dynamics, and encoded Gibbs states at β=2 and β=10. *Right*: The overlap between the quantum state evolved under the Lindblad dynamics and the target Gibbs state, defined as in Eq. **19**. A short evolution time (t≤1) is enough to prepare warm-start states with constant (≥0.2) overlap even at low temperatures.

In Fig. 6, we demonstrate the effectiveness of the Lindbladian-based warm-start preparation method using the same 1D potential as in Fig. 5*A*. We evolve the Lindblad dynamics starting from an initial state with negligible overlap with the encoded Gibbs state (Fig. 6*A*). The overlap between the solution state ρ(t) and the encoded Gibbs state is defined as

[19] $$\begin{array}{c} \sqrt{\text{Tr}\left[|\sqrt{\sigma }\rangle \langle \sqrt{\sigma }|\rho (t)\right]}, \end{array}$$

and Fig. 6*B* illustrates how the overlap changes over time. We observe that a short-time Lindbladian evolution (t=1) already yields a warm-start state with a constant (≥0.2) overlap. Beyond this point, the dynamics requires an evolution time at least an order of magnitude longer to fully converge to the target Gibbs state. This later stage can be significantly accelerated using our quantum-accelerated LD or RELD algorithms.

## Discussion

In this work, we present a framework for accelerating probabilistic sampling using quantum computers. By associating the Gibbs sampling task with the preparation of the encoded Gibbs state, we leverage quantum singular value thresholding to accelerate classical sampling. Our method operates directly at the operator level, without requiring explicit time discretization of stochastic processes, thereby circumventing existing classical algorithms based on simulating discrete-time Markov chains. To the best of our knowledge, this provides the first provable quantum speedup for a broad class of sampling processes, directly in terms of the spectral gap of the infinitesimal generator, which is a type of speedup with no classical counterpart.

Our quantum algorithm relies on preparing the encoded Gibbs state $|\sqrt{\sigma }\rangle$, which corresponds to the stationary state of a Markov chain (i.e., a forward Kolmogorov equation). In the literature, the quantum state corresponding to the stationary distribution of a classical Markov chain is often referred to as a “qsample.” Generating a qsample is a long-standing challenge in quantum computing and is widely regarded as a significantly more powerful task than its classical counterpart (46, 71, 72). In our algorithm, the state $|\sqrt{\sigma }\rangle$ is fully characterized as the ground state of the (generalized) Witten Laplacian H. In fact, given any target probability distribution with density ρ, as long as the quantum state $|\sqrt{\rho_{0}}\rangle$ can be characterized as an eigenstate of a linear operator, a similar singular value thresholding approach may apply to facilitate the sampling from ρ. For example, the ground state of the index-1 Witten Laplacian (the standard Witten Laplacian is also called the index-0 Witten Laplacian) encodes a distribution that locates the metastable configurations over a nonconvex potential function (56, 73). In such cases, our framework may be extended to enable sampling from non-Gibbs distributions, for which few efficient classical algorithms are known.

Our construction of block-encodings for the factor operators $L_{j}$ relies on the quantum implementation of the Fourier transform in $R^{d}$. Extending these techniques to more general manifolds or to nondifferentiable settings remains an open question and an interesting avenue for future research.

In the limit of zero temperature (i.e., β→∞), non-logconcave sampling reduces to a nonconvex optimization problem. Recent works (74–76) propose to leverage quantum dynamics (e.g., Hamiltonian or Lindbladian evolution) for solving nonconvex optimization problems. These dynamics-based quantum optimization algorithms operate independently of classical processes and can exhibit superpolynomial speedups over all classical algorithms for some problem classes (77). This raises a natural question: can certain quantum dynamics be harnessed to achieve large (i.e., superquadratic) acceleration for non-logconcave sampling? If such methods exist, they would greatly expand the design space of quantum algorithms with practical utility.

Quantum Gibbs sampling, which prepares a thermal state $\rho \propto e^{-\beta H}$ for a quantum Hamiltonian H, has seen rapid progress in algorithmic developments and analysis in recent years (78–86). Since classical Gibbs sampling corresponds to the special case where H is diagonal in the computational basis, we hope that the interplay between classical and quantum perspectives may help uncover further quantum advantages in Gibbs sampling tasks.

## Supplementary Material

Appendix 01 (PDF)

## Data Availability

Source code data have been deposited in Github Repository (https://github.com/jiaqileng/operator-level-sampling) (67). All other data are included in the manuscript and/or *SI Appendix*.

## Footnotes

^*^Meanwhile, it is worth noting that the $C_{\mathrm{CG}}^{2}$ dependence in classical MALA is highly technical and may be subject to improvement in future work.

^†^Some literature defines the Witten Laplacian as $4\beta^{-1}H$. Such a constant scaling factor does not affect our analysis.

^‡^Formally, a Hamiltonian $H=\sum_{j}H_{j}$ is frustration-free if the ground state of H is also the ground state for all $H_{j}$.

^§^More precisely, the prepared state |g〉 needs to undergo postprocessing to boost the resolution before measurement in the computational basis, see *SI Appendix*, Lemma 9 for details.

^¶^Due to spatial discretization, the singular value gap in the quantum algorithms can be slightly different from the square root of the Witten Laplacian gap. This error can be exponentially small thanks to the pseudospectral method.
