# Supplementary material for: Operator-level quantum acceleration of non-logconcave sampling
Source: Proc Natl Acad Sci U S A. 2026 Feb 20;123(8):e2512789123. doi: 10.1073/pnas.2512789123 (PMC12933055; doi:10.1073/pnas.2512789123)
Supplement: Supplementary file 1 — Appendix 01 (PDF) [file pnas.2512789123.sapp.pdf]

Supporting Information for

Operator-Level Quantum Acceleration of Non-Logconcave Sampling

Jiaqi Leng, Zhiyan Deng, Zherui Chen, and Lin Lin

Lin Lin
E-mail: linlin math.berkeley.edu

This PDF file includes:

Supporting text
Figs. S1 to S2
SI References

Supporting Information Text

1. Mathematical Preliminaries on Markov Processes

Notation. We consider two types of inner products. The first is the standard L^2 inner product $\langle f, g\rangle := \int_{\mathbb{R}^d} fg\mathrm{d}x$, where we omit the argument x for simplicity. The second is the σ-weighted inner product, defined as $\langle f, g\rangle_\sigma := \int_{\mathbb{R}^d} (fg)\,\sigma\mathrm{d}x$. We also define $\|f\|_{L^2}^2 = \langle f, f\rangle$ and $\|f\|_\sigma^2 = \langle f, f\rangle_\sigma$. For any operator $\mathcal{L}$ acting on a dense subset of $L^2(\mathbb{R}^d)$, let $\mathcal{L}^\dagger$ be the adjoint with respect to the standard L^2 inner product, i.e., $\langle f, \mathcal{L}^\dagger g\rangle = \langle \mathcal{L}f, g\rangle$ for all proper functions f, g.

In this work, for simplicity, we only consider absolutely continuous probability measures that admit a density, and *ergodic* dynamics with a unique fixed point σ. Starting from certain initial distribution $\rho(0)$ from a properly chosen set $\mathcal{S}$, the *mixing time* is defined as:

$$t_{\mathrm{mix}}^{[\cdot]}(\epsilon) = \inf_{t\geq 0} \sup_{\rho(0)\in\mathcal{S}} \{[\cdot]\,(\rho(t),\sigma) \leq \epsilon\}\,. \quad [1]$$

Here, $[\cdot]$ measures the discrepancy between the densities ρ and σ. A standard choice is the total variation (TV) distance (1, 2):

$$\mathrm{TV}(\rho,\sigma) = \frac{1}{2}\int_{\mathbb{R}^d} |\rho(x) - \sigma(x)|\,\mathrm{d}x. \quad [2]$$

Besides the TV distance, $[\cdot]$ can be taken to be the χ^2-divergence (see below), Wasserstein-2 (W_2) distance (3, 4), among others (5–8).

Let $\mathcal{L}$ be the infinitesimal generator of a continuous-time process. We say the process satisfies the detailed balance condition (9, 10) if $\mathcal{L}^\dagger$ is self-adjoint with respect to the σ-weighted inner product, i.e.,

$$\langle f, \mathcal{L}^\dagger g\rangle_\sigma = \langle \mathcal{L}^\dagger f, g\rangle_\sigma. \quad [3]$$

For instance, for the Fokker–Planck equation (Eq. [3] in the main text), we have $\mathcal{L}^\dagger = -\nabla V\cdot\nabla + \beta^{-1}\Delta$. Direct calculation shows that $\mathcal{L}^\dagger$ satisfies the detailed balance condition.

When the detailed balance condition is satisfied, all the eigenvalues of $\mathcal{L}^\dagger$ are real and furthermore non-positive. Since $\mathcal{L}^\dagger(1) = 0$ always holds, the process is ergodic if and only if $\ker(\mathcal{L}^\dagger)$ is one-dimensional (11). For an ergodic dynamics, all other eigenvectors of $\mathcal{L}^\dagger$ are orthogonal to 1 with respect to the σ-weighted inner product. Thus the *spectral gap* of $\mathcal{L}^\dagger$ has the following variational characterization

$$\mathrm{Gap}\left(\mathcal{L}^\dagger\right) := \inf_{f\notin\ker(\mathcal{L}^\dagger)} \frac{\langle f, -\mathcal{L}^\dagger f\rangle_\sigma}{\|f - \int f\sigma\mathrm{d}x\|_\sigma^2}. \quad [4]$$

Define the variance of a function f as $\mathrm{Var}_\sigma(f) := \|f - \int f\sigma\mathrm{d}x\|_\sigma^2$. Let $f(x,t) = \rho(x,t)/\sigma(x)$. Then the discrepancy between $\rho(t)$ and σ can be measured by the variance $\mathrm{Var}_\sigma(f) = \|f-1\|_\sigma^2 = \chi^2(\rho(t),\sigma)$, where $\chi^2(\rho,\sigma) = \|\rho/\sigma - 1\|_\sigma^2$ is called the χ^2-divergence and we have used $\int f\sigma\mathrm{d}x = 1$. Notice that

$$\partial_t \mathrm{Var}_\sigma(f) = 2\langle f-1, \partial_t f\rangle_\sigma = 2\langle f-1, \mathcal{L}(f\sigma)\rangle = -2\langle -\mathcal{L}^\dagger(f), f\rangle_\sigma \leq -2\mathrm{Gap}\left(\mathcal{L}^\dagger\right)\mathrm{Var}_\sigma(f). \quad [5]$$

If the spectral gap is positive, then $\rho(t)$ converges exponentially to σ in χ^2-divergence:

$$\chi^2(\rho(t),\sigma) \leq \exp\left(-2\mathrm{Gap}\left(\mathcal{L}^\dagger\right)t\right)\chi^2(\rho(0),\sigma). \quad [6]$$

The last inequality in Eq. (5) is also called the *Poincaré inequality* with a *Poincaré constant* $C_{\mathrm{PI}} = 1/\mathrm{Gap}\left(\mathcal{L}^\dagger\right)$ (12–14).

The Poincaré inequality immediately leads to the exponential convergence of $\rho(t)$ to the stationary measure σ in χ^2-divergence (15, Theorem 1.2.21):

$$\chi^2(\rho(t),\sigma) \leq e^{-\frac{2}{C_{\mathrm{PI}}}t}\chi^2(\rho(0),\sigma). \quad [7]$$

Therefore, a large spectral gap (i.e., a small Poincaré constant) together with a mild initial χ^2-divergence $\chi^2(\rho(0),\sigma)$ implies a fast convergence (i.e., mixing of the process). On the other hand, a small spectral gap is a strong indicator that the mixing process can be slow.

Related works

In this section, we briefly review previous works on continuous sampling in both quantum and classical literature. For simplicity, we focus on a target distribution $\sigma \propto e^{-V}$, i.e., we set $\beta = 1$. A comparison of different results is drawn in Table 1 in the main text.

Solving continuous sampling problems on a quantum computer is a relatively recent development. The current state-of-the-art approaches are based on quantization of classical sampling algorithms using quantum walks (16–18). Ref. (16) proposes several quantum algorithms based on classical sampling algorithms such as ULA and MALA, and achieves quadratic speedup for logconcave distributions. Specifically, assuming the potential V is γ-strongly convex and a warm start initial state, the complexity of the quantum MALA algorithm is $\mathcal{O}(d^{1/4}/\gamma^{1/2})$ (16). The quantum algorithm developed in Ref. (17) (also termed

quantum MALA) is applicable to a broader class of non-logconcave distributions. Given a non-logconcave distribution with density σ, the Cheeger constant (19, 20) is defined as

$$C_{\mathrm{CG}} = \inf_{A\in\mathbb{R}^d} \frac{\liminf_{h\to 0^+} \frac{1}{h}\int_{A_h\setminus A}\sigma \mathrm{d}x}{\min\left\{\int_A \sigma \mathrm{d}x, \int_{A^c}\sigma \mathrm{d}x\right\}}, \quad [8]$$

where $A_h = \{x\colon \exists y \in A,\ \|x-y\|\le h\}$. Starting from a warm start initial state, the complexity is $\mathcal{O}(d^{1/2}C_{\mathrm{CG}})$. While the Cheeger constant can be lower bounded by the Poincaré constant following Cheeger's inequality $C_{\mathrm{PI}} \le 4C_{\mathrm{CG}}^2$ (20, Theorem 13.10), a Busher type inequality states that when $V(x)$ is smooth and ∇V is L-Lipschitz, C_{PI} also produces an upper bound for C_{CG} as $\min\left\{C_{\mathrm{CG}}/(6\sqrt{L}), C_{\mathrm{CG}}^2/36\right\} \le C_{\mathrm{PI}} \le 4C_{\mathrm{CG}}^2$ ((21, Theorem 5.2)). In particular, for a class of highly nonconvex potentials (such as double well potentials), both C_{CG} and C_{PI} can be large and $C_{\mathrm{PI}} = \widetilde{\Theta}(C_{\mathrm{CG}})$. In this case, our complexity achieves a quadratic improvement in terms of the Poincaré constant compared to the results presented in (17). Ref. (18) mainly focus on improving the low-accuracy quantum sampler and achieves the quantum speed up when applied to optimization.

In Refs. (16, 17), the warm-started initial state can be obtained using annealing techniques (16, Section B.3), (17, Section 3). Specifically, when the potential V is γ-strongly convex, the complexity of Quantum Annealed MALA is $\mathcal{O}(d/\gamma^{1/2})$ (16, Theorem C.7). When the target distribution satisfies the Log-Sobolev inequality with constant C_{LSI} *, the complexity becomes $\mathcal{O}(dC_{\mathrm{LSI}}C_{\mathrm{CG}})$ (17, Theorem 6). The annealing techniques can also be used in our method to achieve similar complexity when the target distribution is logconcave or satisfies the Log-Sobolev inequality.

For comparison, continuous Gibbs samplers on classical computers can be broadly classified into two categories: low-accuracy samplers, whose complexity scales inversely polynomially with the precision ϵ, and high-accuracy samplers, whose complexity scales as $\mathrm{polylog}(1/\epsilon)$. Low-accuracy samplers typically arise from discretizations of stochastic processes whose stationary distributions converge to the target distribution σ, such as overdamped or underdamped Langevin dynamics (23–25). In our work, we focus specifically on high-accuracy samplers; therefore, we omit a detailed review of complexity results for low-accuracy samplers. Interested readers are referred to (15, 26–28) for a detailed review. High-accuracy samplers, such as MALA and MHMC, often incorporate a Metropolis–Hastings correction step (29, 30), which ensures the algorithm is unbiased. This correction step enables the use of longer time steps in the algorithm, which reduces the complexity of the algorithm with respect to the precision parameter (31–37). Most of the analysis of these high-accuracy samplers requires a warm start assumption and/or strongly log-concavity. Assuming warm start assumption and V is γ-strongly convex, MALA achieves ϵ accuracy in TV, $\sqrt{\mathrm{KL}}$, $\sqrt{\chi^2}$, $\sqrt{\gamma}W_2$ distance with complexity scaling as $\widetilde{\mathcal{O}}\left(d^{1/2}/\gamma\right)$, which is proved to be optimal for MALA (35). Relaxing the γ-strongly convex condition to isoperimetric bounds in the case of MALA become more complicated, and the question of deriving analogous results for the Poincaré constant remains open, to the best of our knowledge. For general isoperimetric bounds, instead of considering MALA, (15, Chapter 8.6) prove that using the proximal sampler (38–40), it is possible to achieve ϵ-precision in various distance with complexity scaling as $\widetilde{\mathcal{O}}\left(d^{1/2}C_{\mathrm{ISB}}\right)$, where C_{ISB} is the Log-Sobolev or Poincaré constant. (41, Lemma 6.5) analyzes the complexity of projected MALA, where sampling is constrained to a bounded domain with radius independent of d.

In terms of different assumptions, we note that if a function V is γ-strongly convex, then $\exp(-V)$ has $1/\gamma$-Log-Sobolev constant. In addition, Log-Sobolev inequality and Cheeger's inequality are stronger than Poincaré inequality. Specifically, if a distribution satisfies Log-Sobolev inequality with parameter C_{LSI} then this distribution also satisfies Poincaré inequality with the same constant C_{LSI}. Furthermore, if a distribution satisfies Cheeger's inequality with parameter C_{CG} then this distribution also satisfies Poincaré inequality with the same constant $4C_{\mathrm{CG}}^2$ (19). For the classical samplers, we note that by combining a low-accuracy sampler, based on underdamped Langevin Monte Carlo and a proximal sampler with MALA, and assuming access to a warm stationary point of V, the MALA algorithm in (37, Theorem 5.4) achieves the same scaling with the C_{PI}-Poincaré constant, without requiring a warm start for initialization. Related developments for a particular class of non-convex potentials called Gaussian mixture models can be found in (42, 43).

2. Ground State preparation with $\mathcal{H} = \sum_j L_j^\dagger L_j$

In this section, we introduce a quantum algorithm that prepares the ground state of a quantum Hamiltonian of the form $\mathcal{H} = \sum_j L_j^\dagger L_j$. In our algorithm, this operator $\mathcal{H}$ represents the (spatially discretized) generalized Witten Laplacian.

A. Problem formulation. Suppose that we have an N-by-N operator of the form

$$\mathcal{H} = \sum_{j=1}^{p} L_j^\dagger L_j, \quad [9]$$

where each $L_j : \mathbb{C}^N \to \mathbb{C}^N$ is a complex-valued matrix. Since $\mathcal{H}$ is Hermitian and non-negative, by the spectral theorem, all eigenvalues of $\mathcal{H}$ are non-negative real numbers. We denote the eigenvalues of $\mathcal{H}$ as $0 \le \lambda_1 < \lambda_2 \le \cdots \le \lambda_N$. Moreover, we assume that there is a positive $D > 0$ such that

$$0 \le \lambda_1 < \frac{D}{16}, \quad \lambda_k \ge \frac{9D}{16} \quad \forall k = 1, 2, \ldots \quad [10]$$

* We note that the Log-Sobolev is stronger than the Poincaré inequality; see the remarks under Table 1 in the main text. For logconcave distributions, these inequalities are nearly equivalent (22).

In other words, the spectral gap (i.e., the difference between the first two eigenvalues) of $\mathcal{H}$ is of the order $\Theta(D)$. The eigenvector of $\mathcal{H}$ associated with the smallest eigenvalue λ_1 is referred to as the *ground state* of $\mathcal{H}$, denoted by $|g\rangle$. We can write $\mathcal{H} = \mathbb{L}^\dagger\mathbb{L}$, where the block matrix

$$\mathbb{L} := [L_1^\top, L_2^\top, \ldots, L_d^\top]^\top. \quad [11]$$

Lemma 1. *Suppose the matrix $\mathbb{L}$ has singular values $\{\sigma_k\}_{k=1}^N$, arranged in an ascending order. Then, we have*

$$0 \leq \sigma_1 \leq \frac{\sqrt{D}}{4}, \quad \sigma_k \geq \frac{3\sqrt{D}}{4} \quad \forall k = 2, \ldots, N. \quad [12]$$

Moreover, the right singular vector of $\mathbb{L}$ associated with the σ_1 is $|g\rangle$.

Proof. Suppose that the matrix $\mathbb{L}$ has a singular value decomposition (SVD) as $\mathbb{L} = U\Sigma V^\dagger$, then we have $\mathcal{H} = V\Sigma^2 V^\dagger$. The ground state of $\mathcal{H}$ corresponds to the first column in V, which is the singular vector of $\mathbb{L}$ associated with the smallest singular value. Also, there is an one-to-one correspondence between the singular values of $\mathbb{L}$ and the eigenvalues of $\mathcal{H}$: $\sigma_k = \sqrt{\lambda_k}$. Therefore, Eq. (12) is a direct consequence of Eq. (10). □

In quantum numerical linear algebra, the block-encoded matrix is a standard input model that enables several powerful quantum algorithms, including Quantum Singular Value Transformation (QSVT). We now define the block-encoding of a rectangular matrix $A \in \mathbb{C}^{2^n \times 2^p}$. We assume $n \geq p$ without loss of generality.

Definition 2 (Block-encoding of a rectangular matrix)**.** *Given a matrix $A \in \mathbb{C}^{2^n \times 2^p}$ with $n \geq p$, if we can find $\alpha, \epsilon > 0$, and a unitary matrix $U_A \in \mathbb{C}^{2^{n+m} \times 2^{n+m}}$ such that*

$$\|A - \alpha\left(\langle 0^m| \otimes I_{2^n}\right) U_A \left(|0^m\rangle \otimes I_{2^p}\right)\| \leq \epsilon, \quad [13]$$

then U_A is called an (α, m, ϵ)-block-encoding of A.

Intuitively, the unitary operator U_A encodes the matrix A/α in its upper left corner, up to an additive error ϵ. The parameter α is referred to as the *normalization factor*, which ensures the block-encoded matrix A/α has an operator norm no greater than 1. When the precision of the block encoding can be easily controlled, for simplicity we may set $\epsilon = 0$. In this case, we have $A = \alpha\left(\langle 0^m| \otimes I_{2^n}\right) U_A \left(|0^m\rangle \otimes I_{2^p}\right)$, and U_A is called an (α, m)-block-encoding of A. We will extensively use the block encoding of $\mathbb{L}$.

The goal is to prepare a ground state of $\mathcal{H}$ using a quantum computer. Thanks to the factorization $\mathcal{H} = \mathbb{L}^\dagger\mathbb{L}$, the problem is equivalent to preparing the right singular vector of $\mathbb{L}$ associated with the smallest singular value σ_1. This can be achieved by applying a singular value thresholding (SVT) algorithm to an initial state $|\phi\rangle$ to filter out contributions in $|\phi\rangle$ corresponding to higher singular values of $\mathbb{L}$. When the initial state has an $\Omega(1)$ overlap with the target state $|g\rangle$, we will end up with the desired ground state with a constant success probability. We present the complexity analysis of the quantum algorithm in Appendix C. The singular value thresholding algorithm is implemented by QSVT, which is discussed in the next subsection.

B. Singular value thresholding via QSVT. In this section, we provide a brief introduction to the Quantum Singular Value Transformation (QSVT) algorithm (44) for completeness.

For a matrix $A \in \mathbb{C}^{2^n \times 2^p}$ $(n \geq p)$, we consider its singular value decomposition:

$$A = W\Sigma V^\dagger. \quad [14]$$

The columns of W, V are called the left and right singular vectors of A, respectively. Σ is a $2^n \times 2^p$ matrix with the main diagonal given by the singular values $\{\sigma_1, \cdots, \sigma_{2^p}\}$. When A is given as a block encoding, we must have $\|A\| \leq 1$ and the singular values of A are in the interval $[0, 1]$.

Let $f: \mathbb{R} \to \mathbb{C}$ be a scaler function such that $f(\sigma_j)$ is well-defined for all singular values σ_j, we can define a *right* generalized matrix function:

$$f^\triangleright(A) = Vf(\Sigma)V^\dagger, \quad [15]$$

where $f(\Sigma) = \mathrm{diag}(f(\sigma_1), \ldots, f(\sigma_N))$. Similarly, we can define a *left* matrix function and a *balanced* matrix function induced by f. They will not appear in this work.

When the function f is specified as an even polynomial of degree d, the matrix function $f^\triangleright(A)$ can be implemented on a quantum computer via QSVT. For a square matrix A, QSVT can be implemented following (44, Corollary 11). This result can be generalized to rectangular matrices (see e.g., (45)). The following theorem is adapted from (45, Theorem 2.3).

Theorem 3 (QSVT with even polynomials)**.** *Let $A \in \mathbb{C}^{2^n \times 2^p}$ be encoded by its (α, m)-block-encoding U_A. For an even polynomial $F(x) \in \mathbb{R}[x]$ with degree d and $|F(x)| \leq 1$ for any $x \in [-1, 1]$, we can implement a $(1, m+1)$-block-encoding of $F^\triangleright(A/\alpha)$ using U_A, $U_A^\dagger$, m-qubit controlled NOT, and single-qubit rotation gates for $\mathcal{O}(d)$ times.*

To implement the singular value thresholding algorithm for A, we want to filter out all singular values that are greater than or equal to σ_2. Suppose that $0 \leq \sigma_1 \leq s_1 < s_2 \leq \sigma_2$, and we consider the following rectangular filter function

$$f(x) = \begin{cases} 1, & x \in [-s_1, s_1], \\ 0, & x \in [-1, -s_2] \cup [s_2, 1]. \end{cases} \quad [16]$$

Rectangle functions are commonly used in QSVT, and they can be efficiently approximated by even polynomials with an additive error.

Lemma 4 ((44, Corollary 16)). *Let $\delta, \epsilon \in (0, 1/2)$ and $t \in [-1, 1]$. There exist an even polynomial $P(x) \in \mathbb{R}[x]$ of degree $\mathcal{O}(\delta^{-1} \log(\epsilon^{-1}))$, such that $|P(x)| \leq 1$ for all $x \in [-1, 1]$ and*

$$\begin{cases} P(x) \in [0, \epsilon] & x \in [-1, -t-\delta] \cup [t+\delta, 1], \\ P(x) \in [1-\epsilon, 1] & x \in [-t+\delta, t-\delta]. \end{cases}$$

This approximation is asymptotically optimal in the parameters δ and ϵ (46). In practice, the approximate polynomial can be explicitly constructed via convex optimization based methods (see (47, Section IV)). Based on Lemma 4, we can construct an even polynomial that approximates the filter function f, which leads to an efficient quantum implementation of the singular value thresholding algorithm (44, Theorem 19). In what follows, we provide a generalized version that applies to rectangular matrices, together with a proof illustrating the algorithmic procedure.

Proposition 5 (Singular value thresholding). *Let $A \in \mathbb{C}^{2^n \times 2^p}$ be encoded by its (α, m)-block-encoding U_A. Let σ_1, σ_2 be the first two singular values of A and $0 \leq \sigma_1 \leq s_1 < s_2 \leq \sigma_2$. We denote $s = s_2 - s_1$. Let $f(x)$ be the rectangular filter function given in Eq. (16). We can implement a $(1, m+1, \epsilon)$-block-encoding of the matrix function $f^{\triangleright}(A)$ using U_A, $U_A^{\dagger}$, m-qubit controlled NOT, and single-qubit rotation gates for $\mathcal{O}\left(\alpha s^{-1} \log(\epsilon^{-1})\right)$ times.*

Proof. Note that $f^{\triangleright}(A)$ is equivalent to $f^{\triangleright}_{1/\alpha}(A/\alpha)$, where $f_{1/\alpha}(x)$ is a filter function given in Eq. (16) with s_1 and s_2 scaled by a factor of $1/\alpha$. By choosing $t = (s_1 + s_2)/(2\alpha)$ and $\delta = (s_2 - s_1)/(2\alpha)$ in Lemma 4, we can approximate the filter function $f_{1/\alpha}(x)$ up to an additive error ϵ with an even polynomial $P(x)$ of degree $d = \mathcal{O}(\alpha s^{-1} \log(\epsilon^{-1}))$. Then, by Theorem 3, we can implement a $(1, m+1)$-block-encoding of $P^{\triangleright}(A/\alpha)$ using U_A, $U_A^{\dagger}$, m-qubit controlled NOT, and single-qubit rotation gates for $\mathcal{O}(\alpha s^{-1} \log(\epsilon^{-1}))$ times. This yields is a $(1, m+1, \epsilon)$-block-encoding of $f^{\triangleright}(A)$ since $|P(x) - f_{1/\alpha}(x)| \leq \epsilon$ for all $x \in [-1, 1]$. □

C. Singular value thresholding for ground state preparation.

Theorem 6. *Consider $\mathcal{H} = \mathbb{L}^{\dagger}\mathbb{L}$ satisfying Eq. (10) with $\mathbb{L}$ given by Eq. (11). Suppose that we have access to (i) a (α, m)-block-encoding of $\mathbb{L}$ (denoted by $\boldsymbol{U}$), and (ii) a warm start quantum state $|\phi\rangle$ with constant overlap with the ground state $|g\rangle$ of $\mathcal{H}$, i.e., $|\langle\phi|g\rangle| = \Omega(1)$. Then for any $\varepsilon > 0$, we can prepare a quantum state $|\tilde{g}\rangle$ such that $\|\tilde{g} - g\| \leq \varepsilon$ using $\mathcal{O}(\alpha D^{-1/2} \log(\varepsilon^{-1}))$ queries to $\boldsymbol{U}$ and $\mathcal{O}(1)$ copies of the state $|\phi\rangle$.*

Proof. By Lemma 1, the ground state of $\mathcal{H}$ is the same as the right singular vector of $\mathbb{L}$ associated with the smallest singular value $\sigma_1 \leq \sqrt{D}/4$. Therefore, we can implement a singular value thresholding by filtering out all (right) singular vectors associated with singular values greater than or equal to $3\sqrt{D}/4$. Let $f(x)$ be the rectangular filter function defined in Eq. (16) with $s_1 = \sqrt{D}/4$ and $s_2 = 3\sqrt{D}/4$. We can apply Proposition 5 to construct a $(1, m+1, \epsilon)$-block-encoding of $f_s^{\triangleright}(\mathbb{L})$ with $\mathcal{O}(\alpha D^{-1/2} \log(\epsilon^{-1}))$ queries to $\boldsymbol{U}$. In other words, this quantum circuit block-encodes an approximate projector $\tilde{\Pi}$ such that $\|\tilde{\Pi} - |g\rangle\langle g|\,\| \leq \epsilon$. By applying this quantum circuit to an initial state $\left|0^{m+1}\right\rangle \otimes |\phi\rangle$, we will obtain a quantum state

$$\left|0^{m+1}\right\rangle |\tilde{g}\rangle + |\perp\rangle, \quad [17]$$

where $\tilde{g} = \tilde{\Pi}\,|\phi\rangle$. It is deduced from $|\langle g|\phi\rangle| = \Omega(1)$ that $\|\tilde{g} - g\| \leq \epsilon$ and $\|\tilde{g}\| = \Omega(1)$. Therefore, by post-selecting the quantum state flagged by 0^{m+1} (with success probability equals to $\|\tilde{g}\|^2 = \Omega(1)$), we can obtain an ϵ-approximate ground state $|\tilde{g}\rangle$. □

3. Operator-Level Acceleration of Langevin dynamics: Details

The Witten Laplacian of Langevin dynamics takes the form:

$$\mathcal{H} = \sum_{j=1}^{d} L_j^{\dagger} L_j, \quad L_j := -i\frac{1}{\sqrt{\beta}}\partial_{x_j} - i\frac{\sqrt{\beta}}{2}\partial_{x_j} V \quad \forall j \in [d] = \{1, 2, \ldots, d\}. \quad [18]$$

In this section, we use the expression $\mathcal{H} = \mathbb{L}^{\dagger}\mathbb{L}$ with $\mathbb{L} := [L_1^{\top}, L_2^{\top}, \ldots, L_d^{\top}]^{\top}$.

A. Spatial discretization. For $j = 1, \dots, d$, the operators L_j are unbounded operators defined in the real space $\mathbb{R}^d$. To compute these operators on a quantum computer, we need to perform spatial discretization and map them to finite-dimensional matrices.

Suppose that the Gibbs measure σ has a negligible probability mass outside a box $\Omega = [-a, a]^d \subset \mathbb{R}^d$. For the potential V with sufficient growth rate, we may set $a = \mathcal{O}(\log(d/\epsilon))$.[†] Since the Gibbs measure is effectively zero on the boundary of Ω, we assume the numerical domain Ω is induced with a periodic boundary. For simplicity, we assume the problem is further rescaled and translated to the unit box $\Omega = [0, 1]^d$, which will only incur an $\mathcal{O}(a) = \mathcal{O}(\log(d/\epsilon))$ multiplicative overhead in the normalization factor of the block-encoding of $\mathbb{L}$. Let $\mathcal{M} = \{\boldsymbol{x} = (x_1, \dots, x_d) \colon x_j \in \{0, h, \dots, 1-h\}\}$ be a regular mesh in Ω, where $h = 1/N$ and N is the number of grid points used for each dimension. In what follows, we will refer to a as the *truncation length* and N the *discretization number*.

Discretization of L_j. Each component operator L_j can be represented as a *pseudo-differential* operator

$$L_j(u)(\boldsymbol{x}) = \int_{\mathbb{R}^d} e^{2\pi i \boldsymbol{x}\cdot\boldsymbol{\xi}} a_j(\boldsymbol{x}, \boldsymbol{\xi}) \hat{u}(\boldsymbol{\xi}) d\boldsymbol{\xi}, \quad a_j(\boldsymbol{x}, \boldsymbol{\xi}) = \frac{2\pi}{\sqrt{\beta}} \xi_j - i\frac{\sqrt{\beta}}{2} \partial_{x_j} V(\boldsymbol{x}) \quad \forall j \in [d], \qquad [19]$$

where $a_j(\boldsymbol{x}, \boldsymbol{\xi})$ is called the *symbol* of L_j and $\hat{u}(\boldsymbol{\xi})$ is the Fourier transform of $u(\boldsymbol{x})$. For every $j \in [d]$, the symbol $a_j(\boldsymbol{x}, \boldsymbol{\xi})$ naturally splits into two components, where $2\pi\xi_j$ corresponds to the differential operator $-i\partial_{x_j}$, and $-i\beta\partial_{x_j} V/2$ is a multiplicative operator in the computational basis.

By truncating the Euclidean space $\mathbb{R}^d$ to the numerical domain $\Omega = [0, 1]^d$ and imposing a periodic boundary condition, we can represent the truncated operator as:

$$\hat{L}_j(u)(\boldsymbol{x}) = \mathcal{F}^{-1}\left(\frac{2\pi}{\sqrt{\beta}} \xi_j \mathcal{F}(u)(\boldsymbol{\xi})\right)(\boldsymbol{x}) + \partial_{x_j} V(\boldsymbol{x}) u(\boldsymbol{x}), \quad \boldsymbol{x} \in \Omega = [0, 1]^d. \qquad [20]$$

Here, $\mathcal{F}$ and $\mathcal{F}^{-1}$ represents the Fourier transform and inverse Fourier transform in Ω, respectively:

$$\mathcal{F}(u)(\boldsymbol{\xi}) = \int_{\Omega} u(\boldsymbol{x}) e^{-2\pi i \boldsymbol{\xi}\cdot\boldsymbol{x}} \mathrm{d}\boldsymbol{x}, \quad \xi = (\xi_1, \dots, \xi_d) \in \mathbb{Z}^d$$

$$\mathcal{F}^{-1}(w)(\boldsymbol{x}) = \sum_{\boldsymbol{\xi} \in \mathbb{Z}^d} w(\boldsymbol{\xi}) e^{2\pi i \boldsymbol{\xi}\cdot\boldsymbol{x}}, \quad \boldsymbol{x} \in [0, 1]^d.$$

The Fourier transformation can be approximated by a quadrature over the uniform mesh $\mathcal{M}$, and then carried out via the Discrete Fourier Transform (DFT). We denote $\mathcal{K}$ as an indexing set: $\mathcal{K} = \{\boldsymbol{k} = (k_1, \dots, k_d) \colon 0 \le k_j \le N-1,\ j \in [d]\}$, and the Fourier transform can be approximately computed through the d-dimensional DFT:

$$\mathcal{F}(u)(\boldsymbol{\xi}) \approx \frac{1}{N^{d/2}} \sum_{\boldsymbol{k} \in \mathcal{K}} u(\boldsymbol{x}_{\boldsymbol{k}}) e^{-2\pi i \boldsymbol{x}_{\boldsymbol{k}}\cdot\boldsymbol{\xi}}. \qquad [21]$$

In the 1-dimensional case, DFT is equivalent to the Quantum Fourier Transform (QFT), given by the following unitary operator:

$$U_{\mathrm{FT}} \left|j\right\rangle = \frac{1}{\sqrt{N}} \sum_{k=0}^{N-1} e^{2\pi i k j/N} \left|k\right\rangle, \qquad [22]$$

which can be implemented on a quantum computer using $\mathcal{O}(\log^2(N))$ elementary gates and no ancilla qubits (48). In general, a d-dimensional DFT (denoted by $\mathrm{DFT}_{N,d}$) can be implemented by concatenating d 1-dimensional DFT/QFT:

$$\mathrm{DFT}_{N,d} = \underbrace{\mathrm{DFT}_{N,1} \otimes \cdots \otimes \mathrm{DFT}_{N,1}}_{d \text{ copies}} = U_{\mathrm{FT}}^{\otimes d}, \qquad [23]$$

which can be implemented on a quantum computer using $\mathcal{O}(d \log^2(N))$ elementary gates.

Based on the pseudo-differential operator representation Eq. (20), we can write down the spatial discretization of the operator L_j as an N^d-dimensional matrix $\widetilde{L}_j$:

$$\widetilde{L}_j = \frac{2\pi}{\sqrt{\beta}} \mathrm{DFT}_{N,d}^{\dagger} \left(\Phi_{\boldsymbol{\xi}}^j\right) \mathrm{DFT}_{N,d} - i\frac{\sqrt{\beta}}{2} \Phi_{\boldsymbol{x}}^j, \qquad [24]$$

where $\Phi_{\boldsymbol{\xi}}^j$ and $\Phi_{\boldsymbol{x}}^j$ are diagonal matrices defined as follows:

$$\Phi_{\boldsymbol{\xi}}^j \left|\xi_1, \dots, \xi_d\right\rangle = \xi_j \left|\xi_1, \dots, \xi_d\right\rangle, \quad \Phi_{\boldsymbol{x}}^j \left|x_1, \dots, x_d\right\rangle = \partial_{x_j} V(\boldsymbol{x}) \left|x_1, \dots, x_d\right\rangle.$$

They represent the multiplicative operators corresponding to ξ_j and $\partial_{x_j} V$ in Eq. (20), respectively. Note that the frequency number must be in the range $-N/2 \le \xi_j \le N/2 - 1$ for all $j \in [d]$. As a result, the discretized $\mathbb{L}$ operator is as follows:

$$\widetilde{\mathbb{L}} = [\widetilde{L}_1^{\top}, \dots, \widetilde{L}_d^{\top}]^{\top}. \qquad [25]$$

[†] For example, if $V \ge \gamma\|x\|^2$ for some $\gamma > 0$, then we have $\mathbb{P}_\sigma[x \notin \Omega] \le \frac{1}{Z}\int_{\mathbb{R}^d\setminus\Omega} e^{-\gamma\|x\|^2} \mathrm{d}x = \mathcal{O}(dC^{-a})$, where $C > 1$ independent of d. Therefore, to truncate the Gibbs measure up to an error ϵ, it is sufficient to choose $a = \mathcal{O}(\log(d/\epsilon))$.

Discretization of the encoded Gibbs state. Next, we discuss the kernel of the discretized operator $\widetilde{\mathbb{L}}$. As expected, it should correspond to a discretized encoded Gibbs state. Here, we adopt a standard trigonometric interpolation to characterize the discretization error. For $k = 0, \ldots, N-1$, recall that the quadrature point $x_k = kh = k/N$, and we define the following function:

$$\psi_k(x) = \frac{1}{\sqrt{N}} \sum_{j=-N/2}^{N/2-1} e^{2\pi i j (x - x_k)}, \quad x \in [0,1], \tag{26}$$

which is a trigonometric polynomial satisfying $\psi_k(x_l) = \sqrt{N}\delta_{k,l}$ for any $k, l = 0, \ldots, N-1$. Moreover, it is readily verified that

$$\langle \psi_k, \psi_l \rangle_{L^2} = \delta_{k,l} \quad \forall k, l = 0, \ldots, N-1, \tag{27}$$

where $\langle f, g\rangle_{L^2} = \int \bar{f} g \mathrm{d}x$ represents the standard L^2 inner product, and $\|f\|_{L^2} = \langle f, f\rangle_{L^2}$. In Fig. S1, we demonstrate the real and imaginary parts of the function $\psi_0(x)$ with $N = 32$. Intuitively, the functions ψ_k are continuous interpolations of the "square root" of delta functions, i.e., $\psi_k \approx \sqrt{\delta_{x_k}}$.

For a quantum state $|u\rangle = \sum_{k_1,\ldots,k_d=0}^{N-1} u(k_1, \ldots, k_d) |k_1, \ldots, k_d\rangle$, we define the following interpolation map, which can be regarded as an isometry that embeds $\mathbb{C}^{N^d}$ into $L^2(\Omega)$:

$$I_N |u\rangle = \sum_{k_1,\ldots,k_d=0}^{N-1} u(k_1, \ldots, k_d) \psi_{k_1}(x_1) \ldots \psi_{k_d}(x_d). \tag{28}$$

Let $\left|\widetilde{\sqrt{\sigma}}\right\rangle$ be the right singular vector of $\widetilde{\mathbb{L}}$ associated with the smallest singular value (or equivalently, it is the ground state of the discretized Witten Laplacian $\widetilde{H} = \widetilde{\mathbb{L}}^\dagger \widetilde{\mathbb{L}}$). Through the interpolation Eq. (28), we can quantify the discretization error by analyzing $\left\| I_N \left|\widetilde{\sqrt{\sigma}}\right\rangle - \left|\sqrt{\sigma}\right\rangle \right\|_{L^2}$. This will be made rigorous in Assumption 9.

Moreover, the interpolation also provides a practical recipe for achieving high-accuracy Gibbs sampling. If we directly measure $\left|\widetilde{\sqrt{\sigma}}\right\rangle$ using the computational basis, we would realize a random variable $\tilde{X}$ whose distribution is completely supported on the grid points of $\mathcal{M}$. Since the law of $\tilde{X}$ is a finite mixture of Dirac measures, the TV distance between $\tilde{X}$ and the true Gibbs measure σ is always 1! A naive fix could be to spread the probability mass at each mesh point to its neighborhood (like what we did in the classical post-processing in Lemma 7). In this case, the TV distance decays at a rate $\mathcal{O}(1/N)$ because the law of the new random variable is approximately a "Riemann sum" approximation of σ. This decay rate is unfavorable as it implies that the discretization number has to be $N = \mathcal{O}(1/\epsilon)$, which would inevitably lead to a sampling algorithm with a query complexity $\mathrm{poly}(1/\epsilon)$ (i.e., a low-accuracy sampler). In Lemma 7, we exploit the trigonometric interpolation to achieve high-accuracy sampling with a potentially small discretization number N.

Lemma 7 (Resolution booster)**.** *For a fixed $\epsilon > 0$, suppose that we have access to a state $|g\rangle \in \mathbb{C}^{N^d}$ such that*

$$\left\| I_N |g\rangle - \sqrt{\sigma} \right\|_{L^2} \leq \epsilon/2, \tag{29}$$

where $\left|\sqrt{\sigma}\right\rangle \in L^2(\mathbb{R}^d)$ is the encoded Gibbs state.[‡] *Then, there is a quantum algorithm that outputs a random variable X following the distribution η such that $\mathrm{TV}(\eta, \sigma) \leq \epsilon$ with 1 copy of the state $|g\rangle$, and an additional $d \cdot \mathrm{poly}\log(1/\epsilon)$ elementary gates.*

Proof. Without loss of generality, we assume the $\log_2(N) = q$ is a positive integer, i.e., $|g\rangle$ can be represented by dq qubits. For a given integer $r \geq q$ (the choice of r will be specified later), we consider the following unitary operator:

$$U\colon \left|0^{r-q}\right\rangle \otimes |k\rangle \mapsto \left|\overline{\psi_k}\right\rangle, \quad \forall k = 0, \ldots, 2^q - 1, \tag{30}$$

where

$$\left|\overline{\psi_k}\right\rangle = \frac{1}{\mathcal{N}_k} \sum_{j=0}^{2^r-1} \psi_k(j/2^r) |j\rangle, \quad \mathcal{N}_k = \left(\sum_{j=0}^{2^r-1} |\psi_k(j/2^r)|^2 \right)^{1/2}. \tag{31}$$

This operation can be constructed by performing a discrete Fourier transform on q qubits, "padding" the resulting state with a state $\left|0^{r-q}\right\rangle$, and performing an inverse discrete Fourier transform on the $q + (r-q) = r$ qubits. Applying this argument to every dimension, we can add $d(r-q)$ ancilla qubits to the original register that stores $|g\rangle$ and apply $U^{\otimes d}$ to the extended register. The resulting quantum state reads:

$$U^{\otimes d} \left|0^{d(r-q)}\right\rangle \otimes |g\rangle = \sum_{k_1,\ldots,k_d=0}^{N-1} g(k_1, \ldots, k_d) \left|\overline{\psi_{k_1}}\right\rangle \ldots \left|\overline{\psi_{k_d}}\right\rangle, \tag{32}$$

‡ Note that the interpolation function $I_N |g\rangle$ is a function defined in $\Omega \subset \mathbb{R}^d$. It can be naturally extended to the whole space with the same L^2-norm.

which can be regarded as a spatial discretization of $I_N |g\rangle$ with discretization number $M = 2^r$.

Now, by measuring the quantum state $U^{\otimes d} |g\rangle$ using the computational basis, we will obtain a random variable with M^d possible outcomes. These outcomes can be identified with a finer regular mesh in Ω with M^d quadrature points. Then, for an outcome $\mathbf{z} = (z_1, \ldots, z_d)$ (where $z_k \in \{0, 1/2^r, \ldots, 1 - 1/2^r\}$ for $k \in [d]$), we uniformly sample a point in the box centered at $\mathbf{z}$ with an edge length $1/M$. Following this protocol, we realize a random variable $X \in \Omega$ whose probability density $\eta(x)$ is a piece-wise constant function. In particular, this η can be regarded as a "Riemann sum" approximation of the function $\tilde{\sigma} \coloneqq |I_N |u\rangle|^2$. Therefore, by choosing $M = \mathrm{poly}(1/\epsilon)$, we can have

$$\int_\Omega |\eta(x) - \tilde{\sigma}(x)| \,\mathrm{d}x \leq \epsilon. \qquad [33]$$

Moreover, by applying Cauchy-Schwarz inequality to Eq. (29), we have

$$\int |\tilde{\sigma}(x) - \sigma(x)| \,\mathrm{d}x \leq \left\| I_N |u\rangle + \sqrt{\sigma} \right\|_{L^2} \left\| I_N |u\rangle - \sqrt{\sigma} \right\|_{L^2} \leq \epsilon. \qquad [34]$$

Combining Eq. (33) and Eq. (34) using the triangle inequality, we obtain the desired estimate on the TV distance:

$$\mathrm{TV}(\eta, \sigma) = \frac{1}{2} \int |\eta(x) - \sigma(x)| \,\mathrm{d}x \leq \frac{\epsilon}{2} + \frac{\epsilon}{2} = \epsilon. \qquad [35]$$

Note that gate complexity of $U^{\otimes d}$ is $\mathcal{O}(d\,\mathrm{poly}(r)) = d \cdot \mathrm{poly}\log(1/\epsilon)$, where $r = \log_2(M) = \log_2(\mathrm{poly}(1/\epsilon))$. □

Remark 8. *A similar interpolation technique has been introduced in (49, Theorem 3.2) to exponentially improve the sampling resolution. To ensure the Gibbs measure can be efficiently interpolated using a slightly different set of trigonometric polynomials, (49) requires the target Gibbs measure σ to be "semi-analytical", and the purpose is similar to our Assumption 9, as detailed below.*

For a smooth V with a sufficient growth rate, the state $\sqrt{\sigma}$ is also smooth and has a fast-decaying tail. In this case, the DFT-based discretization (also known as a pseudo-spectral method in numerical analysis) often exhibits *spectral convergence*, i.e., the discretization error decays super-polynomially fast with an increasing discretization number N. In this paper, we make the following assumption that ensure the efficiency of the spatial discretization of Langevin dynamics:

Assumption 9 (Spatial discretization of Langevin dynamics)**.** *Let $V\colon \mathbb{R}^d \to \mathbb{R}$ be a smooth potential function, and $\sigma \propto e^{-\beta V}$ be the Gibbs measure. For an arbitrary $\epsilon > 0$, we assume that we can choose $a = \mathcal{O}(\log(d/\epsilon))$ and $N = a \cdot \mathrm{poly}\log(d/\epsilon)$ such that the followings hold:*

1. *The discretized Witten Laplacian $\widetilde{\mathcal{H}} \coloneqq \sum_{j=1}^d \widetilde{L}_j^\dagger \widetilde{L}_j$ has a ground state $\left|\widetilde{\sqrt{\sigma}}\right\rangle$ that satisfies $\left\| I_N \left|\widetilde{\sqrt{\sigma}}\right\rangle - \sqrt{\sigma} \right\|_{L^2} \leq \epsilon/4$,*
2. *Compared to the Witten Laplacian $\mathcal{H}$ in Eq. (18), the smallest eigenvalue of $\widetilde{\mathcal{H}}$ is no greater than $\mathrm{Gap}(\mathcal{H})/16$, and the second smallest eigenvalue of $\widetilde{\mathcal{H}}$ is no smaller than $9\mathrm{Gap}(\mathcal{H})/16$.*

We numerically implemented our DFT-based discretization scheme, and the results suggest that such a discretization scheme achieves an accuracy ϵ with only poly-logarithmically large truncation length a and discretization number N for several non-convex potentials.

B. Block-encoding of $\mathbb{L}$. In this subsection, we discuss how to efficiently block-encode the operator $\mathbb{L}$ using a quantum computer. We assume access to the gradient (i.e., first-order) oracle of V:

$$O_{\nabla V}\colon\ |\boldsymbol{x}\rangle |\boldsymbol{z}\rangle \mapsto |\boldsymbol{x}\rangle |\nabla V(\boldsymbol{x}) + \boldsymbol{z}\rangle = |\boldsymbol{x}\rangle |\partial_1 V(\boldsymbol{x}) + z_1, \ldots, \partial_d V(\boldsymbol{x}) + z_d\rangle$$

and its inverse $O_{\nabla V}^\dagger$. Each sub-register (i.e, $|\boldsymbol{x}\rangle$ and $|\boldsymbol{z}\rangle$, respectively) consists of bd qubits, where b is the number of bits for the fixed point number representation and d is the number of components in the vector. For simplicity, we assume b is a fixed constant (e.g., 32), and the problem dimension d is a *power of* 2, i.e., $\log_2(d)$ is a positive integer.

The block-encoding of each $\widetilde{L}_j$ is based on the spatial discretization Eq. (24), which requires us to block-encode the following matrices:

$$\tilde{P}_j = \frac{2\pi}{\sqrt{\beta}} \left(U_{\mathrm{FT}}^\dagger\right)^{\otimes d} \left(\Phi_{\boldsymbol{\xi}}^j\right) U_{\mathrm{FT}}^{\otimes d}, \quad \tilde{Q}_j = -i\frac{\sqrt{\beta}}{2} \Phi_{\boldsymbol{x}}^j. \qquad [36]$$

Lemma 10 (Select oracle)**.** *Let*

$$\mathrm{SEL} = \sum_{j=1}^d |j\rangle\langle j| \otimes U_{\tilde{P}_j},$$

where $U_{\tilde{P}_j}$ is a $(\pi N/\sqrt{\beta}, 1, 0)$-block-encoding of $\tilde{P}_j$. We can implement the unitary SEL *with $d \cdot \mathrm{poly}\log(d, N)$ elementary gates.*

Proof. The block-encoding of the select oracle can be implemented as follows. We consider the following register:

$$\underbrace{|000\dots0\rangle}_{\text{dim. index}} \otimes \underbrace{|\boldsymbol{\xi}\rangle}_{\text{frequency number}} \otimes \underbrace{|0\rangle}_{\text{ancilla}}. \quad [37]$$

The first sub-register (for dimension indexing) consists of $\log_2(d)$ qubits[§], the second sub-register (for frequency number) consists of db qubits, and the last (ancilla) sub-register has 1 qubit.

To compute the diagonal differential coefficient matrix $\Phi^j_{\boldsymbol{\xi}}$, we can implement a sequence of b controlled rotations, each controlled on the index sub-register, and perform a rotation on the ancilla qubit. These controlled rotations use classical arithmetic operators[¶] to compute the normalized frequency $2\xi_j/N \in [-1, 1]$, and can be executed by $\mathrm{poly}(b) \cdot \log(d)$ elementary gates, where $\mathrm{poly}(b)$ is the cost for implementing classical arithmetic operators, and $\log(d)$ is the overhead for the controlled rotation on $|j\rangle$ basis. We obtain the following state:

$$|j\rangle \otimes |\boldsymbol{\xi}\rangle \otimes |0\rangle \mapsto |j\rangle \otimes |\boldsymbol{\xi}\rangle \otimes \left(\frac{\xi_j}{N/2}|0\rangle + \sqrt{1 - \frac{\xi_j^2}{(N/2)^2}}|1\rangle\right). \quad [38]$$

In other words, this implements a $(N/2, 1)$-block-encoding of $|j\rangle\langle j| \otimes \tilde{\Phi}^j_{\boldsymbol{\xi}_j}$. By sandwiching this unitary operator between the d-dimensional QFT and its inverse, we obtain a controlled unitary $U'_{\tilde{P}_j} = |j\rangle\langle j| \otimes U_{\tilde{P}_j} + \sum_{k\neq j} |k\rangle\langle k| \otimes I$, where $U_{\tilde{P}_j}$ is a $(\pi N/\sqrt{\beta}, 1)$-block-encoding of $\tilde{P}_j$. Note that the d-dimensional QFT (or its inverse) can be implemented using $\mathcal{O}(d \log^2(N))$ elementary qubits. By concatenating d such controlled unitaries in a sequence, we can implement the select oracle SEL with 1 ancilla qubit and $d \cdot \mathrm{poly}\log(d, N)$ elementary gates. □

Let $\mathcal{A} \in \mathbb{C}^{dN^d \times dN^d}$ be the following block matrix:

$$\mathcal{A} = [\tilde{L}_1^\top, \tilde{L}_2^\top, \dots, \tilde{L}_d^\top]^\top \quad [39]$$

We can write $\mathcal{A} = \mathcal{A}_1 + \mathcal{A}_2$ with ($\tilde{P}_j$, $\tilde{Q}_j$ are the same as in Eq. (36))

$$\mathcal{A}_1 = [\tilde{P}_1^\top, \tilde{P}_2^\top, \dots, \tilde{P}_d^\top]^\top, \quad \mathcal{A}_2 = [\tilde{Q}_1^\top, \tilde{Q}_2^\top, \dots, \tilde{Q}_d^\top]^\top.$$

Lemma 11. *We can implement a $(\pi N\sqrt{d/\beta}, 1)$-block-encoding of the matrix $\mathcal{A}_1$ with $d \cdot \mathrm{poly}\log(d, N)$ elementary gates.*

Proof. Recall that we assume the problem dimension d is a power of 2. Therefore, we can prepare a uniform superposition state $|\Psi\rangle = \frac{1}{\sqrt{d}}\sum_{j=0}^{d-1}|j\rangle$ using $\log_2(d)$ Hadamard gates. Then, by applying the layer of Hadamard gates followed by the select oracle SEL, we prepared a $(\pi N\sqrt{d/\beta}, 1)$-block-encoding of $\mathcal{A}_1$. The total number of elementary gates is $d \cdot \mathrm{poly}\log(d, N)$. □

Remark 12. *Note that the $\mathcal{O}(\sqrt{d})$ overhead in the normalization factor of the block-encoding of $\mathcal{A}_1$ is inevitable because $\|\mathcal{A}_1\| = \Omega(\sqrt{d})$.*

Definition 13. *A differentiable function $V\colon \mathbb{R}^d \to \mathbb{R}$ is ℓ-smooth if for any $x, y \in \mathbb{R}^d$,*

$$\|\nabla V(x) - \nabla V(y)\| \leq \ell\|x - y\|,$$

where $\|\cdot\|$ stands for the standard Euclidean distance in $\mathbb{R}^d$. In other words, V is ℓ-smooth if its gradient is ℓ-Lipschitz continuous.

In the following lemma, we show that a $(\sqrt{\beta}R/2, 1)$-block-encoding of $\mathcal{A}_2$ can be constructed using 2 queries to $O_{\nabla V}$. Here, $R = \max_{\boldsymbol{x}\in\Omega}\|\nabla V(\boldsymbol{x})\|$. For an ℓ-smooth potential V with at least 1 local minimum in the numerical domain Ω, it is clear that $R \leq a\ell\sqrt{d} = \mathcal{O}(\ell\sqrt{d}\log(d/\epsilon))$ because Ω has a diameter $a\sqrt{d} = \mathcal{O}(\sqrt{d}\log(d/\epsilon))$.

Lemma 14. *Let $R = \max_{\boldsymbol{x}\in\Omega}\|\nabla V(\boldsymbol{x})\|$. We can implement a $(\sqrt{\beta}R/2, 1)$-block-encoding of $\mathcal{A}_2$ with 2 queries to the gradient oracle $O_{\nabla V}$ or its inverse, and an additional $\tilde{\mathcal{O}}(d^2)$ elementary gates.*

Proof. We introduce an indexing register with d qubits (i.e., "dim. index") to achieve this goal. Consider the following register:

$$\underbrace{|0^{\otimes m_1}\rangle}_{\text{dim. index}} \otimes \underbrace{|\boldsymbol{x}\rangle}_{\text{mesh point}} \otimes \underbrace{|0^{\otimes bd}\rangle}_{\text{anc. grad.}} \otimes \underbrace{|0\rangle}_{\text{ancilla}}. \quad [40]$$

The sizes of the sub-registers are: $m_1 = \lceil\log_2(d)\rceil$, $d\log_2(N)$, bd (recall that b is fixed bit-width precision), and 1. First, we apply the oracle $O_{\nabla V}$ on the second and the third sub-register, and the state becomes:

$$|0^{\otimes m_1}\rangle \otimes |\boldsymbol{x}\rangle \otimes |\partial_1 V(\boldsymbol{x}), \partial_2 V(\boldsymbol{x}), \dots, \partial_d V(\boldsymbol{x})\rangle \otimes |0\rangle. \quad [41]$$

[§] Here, we assume d is a power of 2 so all computational basis in the indexing register is used.

[¶] Any classical arithmetic operations expressed as logical circuits can be implemented on a quantum computer using the same number of elementary gates, see (50, Chapter 4).

Next, we consider a sequence of d "controlled" rotation gadgets, while using the first register as a counting register. The first rotation is controlled on the third register and rotates the basis in the (combined) first and last sub-register:

$$|0^{m_1}\rangle \otimes |0\rangle \mapsto \left(\frac{\partial_1 V(\boldsymbol{x})}{R}|0\rangle|0\rangle + \frac{\sqrt{R^2 - |\partial_1 V(\boldsymbol{x})|^2}}{R}|0\rangle|1\rangle\right). \quad [42]$$

Note that the $|1\rangle|0\rangle$ state in the right-hand side of Eq. (42) represents the tensor product of $|1\rangle$ in the first sub-register and $|0\rangle$ in the last sub-register. The interpretation of $|0\rangle|1\rangle$ and other product states that follow is similar. In other words, this is a controlled Givens rotation operation (i.e., a rotation acting on 2 entries in the computational basis) that encodes the first partial derivative $\partial_1 V(\boldsymbol{x})$ to the basis $|0\rangle|0\rangle$ and the remainder in the basis $|0\rangle|1\rangle$. This rotation gadget can be efficiently implemented by controlling on the first b qubits in the third sub-register.

The second rotation is again controlled on the gradient sub-register and acts non-trivially on two basis states:

$$|0\rangle|1\rangle \mapsto \sin(\theta)|0\rangle|1\rangle + \cos(\theta)|1\rangle|0\rangle, \quad [43]$$

where

$$\sin(\theta) = \frac{\sqrt{R^2 - |\partial_1 V(\boldsymbol{x})|^2 - |\partial_2 V(\boldsymbol{x})|^2}}{\sqrt{R^2 - |\partial_1 V(\boldsymbol{x})|^2}}, \quad \cos(\theta) = \frac{\partial_2 V(\boldsymbol{x})}{\sqrt{R^2 - |\partial_1 V(\boldsymbol{x})|^2}}. \quad [44]$$

By applying this rotation to the state Eq. (42), we encode the second partial derivative $\partial_2 V$ to the amplitude of $|1\rangle|0\rangle$:

$$\left(\frac{\partial_1 V(\boldsymbol{x})}{R}|0\rangle|0\rangle + \frac{\partial_2 V(\boldsymbol{x})}{R}|1\rangle|0\rangle + \frac{\sqrt{R^2 - |\partial_1 V(\boldsymbol{x})|^2 - |\partial_2 V(\boldsymbol{x})|^2}}{R}|0\rangle|1\rangle\right). \quad [45]$$

It is worth noting that the rotation angle has to be carefully computed (via classical arithmetic circuits) to keep track of the previous subnormalization factors, which requires reading the first $2b$ qubits in the third sub-register.

Iterating this process for all $|j\rangle|0\rangle$ basis for $j = 0, \ldots, d-1$, we will prepare a quantum state that encodes the j-th partial derivative of V in the amplitude of the computational basis $|j-1\rangle|0\rangle$. Since each Givens rotation has to compute the subnormalization factor using all previous gradients, the overall number of operations scales as $1 + 2 + \cdots + d = \mathcal{O}(d^2)$. Finally, we apply the inverse of $O_{\nabla V}$ to uncompute the third register (i.e., $|\nabla V(\boldsymbol{x})\rangle$) and discard it thereafter. The resulting state is

$$\sum_{j=1}^{d} \frac{\partial_j V(\boldsymbol{x})}{R}|j\rangle|\boldsymbol{x}\rangle|0\rangle + |\perp\rangle, \quad [46]$$

which implements a $(\sqrt{\beta}R/2, 1)$-block-encoding of $\mathcal{A}_2$. □

Finally, we can perform LCU to construct a block-encoding of $\mathcal{A}$ (the discretized $\mathbb{L}$).

Proposition 15 (Block-encoding of $\mathbb{L}$)**.** *Let N and R are the same as above, and $\alpha = \pi N\sqrt{d/\beta} + \sqrt{\beta}R/2$. We can implement an $(\alpha, 3)$-block-encoding of the matrix $\mathcal{A}$ with 2 queries to the gradient oracle $O_{\nabla V}$ (or its inverse) and an additional $\widetilde{\mathcal{O}}(d^2)$ elementary gates. Here, the $\widetilde{\mathcal{O}}(\cdot)$ notation suppresses poly-logarithmic factors in d and N.*

Proof. Note that $\mathcal{A} = \mathcal{A}_1 + \mathcal{A}_2$, and the block-encoding of $\mathcal{A}_1$ and $\mathcal{A}_2$ can be constructed using Lemma 11 and Lemma 14, respectively. Effectively, they can be regarded as block-encodings of $\mathcal{A}_1/\alpha_1$ and $\mathcal{A}_2/\alpha_2$, each with subnormalization factors 1. Now, we invoke the Linear Combination of Unitaries (LCU) technique (44, Lemma 29) to construct a block-encoding of $\mathcal{A}$. Due to the different subnormalization factors, we use an LCU coefficient pair $y = (\alpha_1/(\alpha_1+\alpha_2), \alpha_2/(\alpha_1+\alpha_2))$. As a result, we obtain a $(\|y\|_1, 3)$-block-encoding of

$$y_1\left(\frac{\mathcal{A}_1}{\alpha_1}\right) + y_2\left(\frac{\mathcal{A}_2}{\alpha_2}\right) = \frac{\mathcal{A}}{(\alpha_1+\alpha_2)}, \quad [47]$$

which corresponds to a block-encoding of $\mathcal{A}$ with a normalization factor

$$\alpha = \pi N\sqrt{d/\beta} + \sqrt{\beta}R/2. \quad [48]$$

Note that the number of ancilla qubits in the block-encoding of $\mathcal{A}$ is 3 since $\mathcal{A}_1$ and $\mathcal{A}_2$ each require 1 ancilla qubit, and LCU requires an additional ancilla qubit. □

C. Proof of Theorem 1. The following result is a rigorous version of Theorem 1.

Theorem 16. *Suppose that Assumption 9 holds and the potential V is ℓ-smooth. Assume access to a state $|\phi\rangle$ (i.e., warm start) such that $\left|\left\langle \phi \middle| \widetilde{\sqrt{\sigma}} \right\rangle\right| = \Omega(1)$. Then, there exists a quantum algorithm that outputs a random variable $X \in \mathbb{R}^d$ distributed following a probability distribution η such that $\mathrm{TV}(\eta, \sigma) \leq \epsilon$ using*

$$\sqrt{\beta d C_{\mathrm{PI}}} \cdot \left(\beta^{-1} + \ell\right) \cdot \mathrm{poly}\log\left(d, \epsilon^{-1}\right) \quad [49]$$

quantum queries to the gradient ∇V, and $\mathcal{O}(1)$ copies of the state $|\phi\rangle$.

Proof. For $j = 1, \ldots, d$, we denote $\widetilde{L}_j$ as the spatially discretized L_j as given in Eq. (24). We write $\widetilde{\mathbb{L}} := [\widetilde{L}_1^\top, \widetilde{L}_2^\top, \ldots, \widetilde{L}_d^\top]^\top$. By Definition 2, the block-encoding of $\mathcal{A}$ constructed in Proposition 15 provides a $(\alpha, 3)$-block-encoding of $\widetilde{\mathbb{L}}$ with the normalization factor $\alpha = \pi N\sqrt{d/\beta} + \sqrt{\beta}R/2$. For a fixed accuracy parameter $\epsilon > 0$, due to Assumption 9, we can truncate the numerical domain to $\Omega = [-a, a]^d$ with an edge length $a = \mathcal{O}(\log(d/\epsilon))$, which implies that the diameter of Ω is $\mathcal{O}(\sqrt{d}\log(d/\epsilon))$. Then, by the ℓ-smoothness of V, we have $R = \max_{x\in\Omega} \|\nabla V(x)\| = \mathcal{O}(\ell\sqrt{d}\log(d/\epsilon))$. It follows that

$$\alpha = \pi N\sqrt{d/\beta} + \sqrt{\beta}R/2 = \mathcal{O}\left(N\sqrt{\beta d} \cdot \log(d/\epsilon) \cdot (\beta^{-1} + \ell)\right). \quad [50]$$

Recall that the discretized Witten Laplacian $\widetilde{\mathcal{H}} = \sum_{j=1}^d \widetilde{L}_j^\dagger \widetilde{L}_j$ has a unique ground state $\left|\widetilde{\sqrt{\sigma}}\right\rangle$. Given access to a warm start state $|\phi\rangle$ such that $\left|\left\langle \phi \middle| \widetilde{\sqrt{\sigma}} \right\rangle\right| = \Omega(1)$, by Theorem 6, we can prepare a quantum state $|g\rangle$ such that $\left\|g - \widetilde{\sqrt{\sigma}}\right\| \leq \epsilon/4$ with

$$\mathcal{O}\left(\frac{\alpha}{\sqrt{\mathrm{Gap}\,(\mathcal{H})}}\right) = \sqrt{\beta d C_{\mathrm{PI}}} \cdot (\beta^{-1} + \ell) \cdot \mathrm{poly}\log\left(d, \epsilon^{-1}\right) \quad [51]$$

queries to the block-encoding of $\mathcal{A}$, which amounts to the same query complexity to $O_{\nabla V}$ due to Proposition 15. Since the success probability is $\Omega(1)$ due to warm start, the total number of copies of $|\phi\rangle$ required is $\mathcal{O}(1)$. By Assumption 9, $\left\|I_N \left|\widetilde{\sqrt{\sigma}}\right\rangle - \sqrt{\sigma}\right\|_{L^2} \leq \epsilon/4$ and I_N is an isometry. Thus, due to the triangle inequality,

$$\left\|I_N |g\rangle - \sqrt{\sigma}\right\|_{L^2} \leq \epsilon/2. \quad [52]$$

Now, by invoking Lemma 7, we can realize a random variable $X \sim \eta$ such that $\mathrm{TV}(\eta, \sigma) \leq \epsilon$ with the number of queries to ∇V given by Eq. (49). □

Remark 17. *For logconcave sampling, our query complexity exhibits scaling invariance with respect to the temperature $T = 1/\beta$. In particular, when the potential V is γ-strongly convex, the Poincaré constant always reads $C_{\mathrm{PI}} = 1/\gamma$. Then, the query complexity of our quantum algorithm is*

$$\sqrt{\frac{d}{\beta\gamma}} \cdot (1 + \ell\beta) \cdot \mathrm{poly}\log\left(d, \epsilon^{-1}\right). \quad [53]$$

Remark 18. *For the warm start condition, we have two comments:*

- *Our warm start condition $\left|\left\langle \sqrt{\rho_0} \middle| \sqrt{\sigma} \right\rangle\right| = \Omega(1)$ does not require that ρ_0 has mass on every region where σ does. For example, let $\sigma(x)$ be a mixture of two Gaussians: $\sigma(x) \propto \exp\left(-|x|^2/(2R^2)\right) + \exp\left(-|x - a|^2/(2R^2)\right)$, and $\rho(x) \propto \exp\left(-|x|^2/(2R^2)\right)$. When $R \ll 1$, we have $\left|\left\langle \sqrt{\sigma} \middle| \sqrt{\rho_0} \right\rangle\right| \approx 1/2 = \Omega(1)$. However, it is straightforward to see that $\sigma(B_a(R)) = \Omega(1)$ and $\rho_0(B_a(R)) \ll 1$, showing that ρ_0 does not "cover" the entire support of σ.*

- *We note that the warm-start assumption in (16) is strictly stronger than ours. In (16, (C.15)), it is assumed that the initial state $\left|\sqrt{\rho_0}\right\rangle \in L^2(\mathbb{R}^d)$ satisfying $\|\rho_0\|_{L^2} = 1$ is β-warm, i.e.,*

$$\sup_x \rho_0(x)/\sigma(x) \leq \beta, \quad [54]$$

  where $\beta > 0$ is a constant independent of C_{PI} and d. This condition immediately implies our warm start assumption: for a β-warm initial state $|\psi\rangle$, we have

$$\left\langle \sqrt{\rho_0} \middle| \sqrt{\sigma} \right\rangle = \int_{\mathbb{R}^d} \sqrt{\rho_0(x)\sigma(x)}\ \mathrm{d}x \geq \beta \int_{\mathbb{R}^d} \rho_0\ \mathrm{d}x = \beta = \Omega(1). \quad [55]$$

  Furthermore, we note that it suffices to assume

$$\min\left\{\sup_x \frac{\rho_0(x)}{\sigma(x)}, \sup_x \frac{\sigma(x)}{\rho_0(x)}\right\} \leq \beta, \quad [56]$$

  in order to guarantee that $\left\langle \sqrt{\rho_0} \middle| \sqrt{\sigma} \right\rangle$ is at least β. As a concrete example, let $\rho_0(x)$ be a mixture of two Gaussians: $\rho_0(x) \propto \exp\left(-|x|^2/(2R^2)\right) + \exp\left(-|x - a|^2/(2R^2)\right)$, and let $\rho_1(x) \propto \exp\left(-|x|^2/(2R^2)\right)$. When $R \ll 1$, we have $\left|\left\langle \sqrt{\rho_0} \middle| \sqrt{\rho_1} \right\rangle\right| \approx 1/2$, even though ρ_0 is not a constant-warm start initial state for ρ_1 according to Eq. (54), as the warmness parameter $\beta = \Omega\left(\exp\left(|a|^2/R^2\right)/R^d\right)$ becomes exponentially large as $R \to 0$.

4. Operator-Level Acceleration of Replica Exchange: Details

A. Derivation of the generalized Witten Laplacian. In this section, we derive the generalized Witten Laplacian for replica exchange Langevin diffusion (RELD). Recall that, for two inverse temperatures $\beta > \beta' > 0$, the forward Kolmogorov equation of RELD is given by

$$\begin{aligned}\partial_t \rho = \mathcal{L}(\rho) \coloneqq & \underbrace{\nabla_x \cdot (\nabla_x V(x)\rho(t,x,y)) + \beta^{-1}\Delta_x \rho(t,x,y)}_{\mathcal{L}_1(\rho(t,x,y))} + \underbrace{\nabla_y \cdot (\nabla_y V(x)\rho(t,x,y)) + \beta'^{-1}\Delta_y \rho(t,x,y)}_{\mathcal{L}_2(\rho(t,x,y)}\\ & + \underbrace{\mu\left(s(y,x)\rho(t,y,x) - s(x,y)\rho(t,x,y)\right)}_{\mathcal{L}_s(\rho(t,x,y))}.\end{aligned} \quad [57]$$

The first two operators $\mathcal{L}_1$ and $\mathcal{L}_2$ correspond to the low- and high-temperature Langevin dynamics, respectively. The third operator $\mathcal{L}_s$ corresponds to a Metropolis-Hasting type swapping operations between the two continuous-time Markov chains, with the swapping probability given by

$$s(x,y) = \min\left(1, \frac{\boldsymbol{\sigma}(y,x)}{\boldsymbol{\sigma}(x,y)}\right). \quad [58]$$

For more details on the generator of the swapping mechanism ($\mathcal{L}_s$), we refer the readers to (43).

The generalized Witten Laplacian of RELD is obtained via the similarity transformation

$$\mathcal{H} = -\boldsymbol{\sigma}^{-1/2} \circ \mathcal{L} \circ \boldsymbol{\sigma}^{1/2},$$

where the joint Gibbs measure is the invariant measure of RELD dynamics:

$$\boldsymbol{\sigma}(x,y) \propto \exp\left(-\beta V(x) - \beta' V(y)\right).$$

We can compute the generalized Witten Laplacian for each component operator in $\mathcal{L}$. When applying the similarity transformation to the operator $\mathcal{L}_1$, we obtain the Witten Laplacian corresponding to the low-temperature Langevin dynamics:

$$\mathcal{H}_1 = -\boldsymbol{\sigma}^{-1/2} \circ \mathcal{L}_1 \circ \boldsymbol{\sigma}^{1/2} = -\frac{1}{\beta}\Delta_x + \left(\frac{\beta}{4}\|\nabla_x V\|^2 - \frac{1}{2}\Delta_x V\right). \quad [59]$$

The similarity transformation also maps $\mathcal{L}_2$ to the Witten Laplacian of the high-temperature Langevin dynamics:

$$\mathcal{H}_2 = -\boldsymbol{\sigma}^{-1/2} \circ \mathcal{L}_2 \circ \boldsymbol{\sigma}^{1/2} = -\frac{1}{\beta'}\Delta_y + \left(\frac{\beta'}{4}\|\nabla_y V\|^2 - \frac{1}{2}\Delta_y V\right). \quad [60]$$

These are frustration-free Hamiltonians and can be factorized in the same way as the standard Witten Laplacian:

$$\mathcal{H}_1 = \sum_{j=1}^{d} L_j^\dagger L_j, \quad L_j \coloneqq -i\frac{1}{\sqrt{\beta}}\partial_{x_j} - i\frac{\sqrt{\beta}}{2}\partial_{x_j} V, \quad [61]$$

$$\mathcal{H}_2 = \sum_{j=1}^{d} L_j^{'\dagger} L_j', \quad L_j' \coloneqq -i\frac{1}{\sqrt{\beta'}}\partial_{y_j} - i\frac{\sqrt{\beta'}}{2}\partial_{y_j} V. \quad [62]$$

The similarity transformation of the swap operator $\mathcal{L}_s$ is more involved and has been discussed in the main text (see Eq. [15]). The corresponding self-adjoint operator reads:

$$\mathcal{H}_s \coloneqq -\boldsymbol{\sigma}^{-1/2} \circ \mathcal{L}_s \circ \boldsymbol{\sigma}^{1/2} = L_s^\dagger L_s, \quad L_s = \sqrt{\frac{\mu}{2}}(I - W)S^{1/2}, \quad [63]$$

where μ is the swapping intensity, S is the point-wise multiplication defined by $S[\psi(x,y)] \coloneqq s(x,y)\psi(x,y)$ with

$$s(x,y) = \min\left(1, \frac{\boldsymbol{\sigma}(y,x)}{\boldsymbol{\sigma}(x,y)}\right) = \exp\left(0 \wedge (\beta - \beta')\cdot(V(x) - V(y))\right), \quad [64]$$

and $W[\psi(x,y)] = \psi(y,x)$ swaps the x and y variables in a test function ψ. It can be readily verified that the encoded Gibbs state $\left|\sqrt{\boldsymbol{\sigma}}\right\rangle$ is annihilated by L_s:

$$\begin{aligned}L_s\left|\sqrt{\boldsymbol{\sigma}}\right\rangle &= \sqrt{\frac{\mu}{2}}(I - W)S^{1/2}\sqrt{\boldsymbol{\sigma}(x,y)}\\ &= \sqrt{\frac{\mu}{2}}(I - W)\left[\sqrt{\boldsymbol{\sigma}(x,y)}1_{A_1} + \sqrt{\boldsymbol{\sigma}(y,x)}1_{A_2}\right]\\ &= \sqrt{\frac{\mu}{2}}\left[\sqrt{\boldsymbol{\sigma}(x,y)}1_{A_1} + \sqrt{\boldsymbol{\sigma}(y,x)}1_{A_2} - \sqrt{\boldsymbol{\sigma}(y,x)}1_{A_2} - \sqrt{\boldsymbol{\sigma}(x,y)}1_{A_1}\right]\\ &= 0,\end{aligned} \quad [65]$$

where $A_1 = \{(x,y) \in \mathbb{R}^{2d} \colon \boldsymbol{\sigma}(y,x) > \boldsymbol{\sigma}(x,y)\}$ and $A_2 = \mathbb{R}^d \backslash A_1$. By symmetry, $W I_{A_1} = I_{A_2}$ and $W I_{A_2} = I_{A_1}$. It follows that the generalized Witten Laplacian of RELD can be written as

$$\mathcal{H} = \mathbb{L}_{\mathrm{RE}}^\dagger \mathbb{L}_{\mathrm{RE}}, \quad [66]$$

where $\mathbb{L}_{\mathrm{RE}}$ is a block matrix consisting of $(2d+1)$ operators:

$$\mathbb{L}_{\mathrm{RE}} = [L_1^\top, \dots, L_d^\top, {L'_1}^\top, \dots, {L'_d}^\top, L_s^\top]^\top. \quad [67]$$

Since the generalized Witten Laplacian $\mathcal{H}$ is frustration-free and the encoded Gibbs state $\left|\sqrt{\boldsymbol{\sigma}}\right\rangle$ is annihilated by all operators L_j, L'_j, and L_s. It is clear that $\left|\sqrt{\boldsymbol{\sigma}}\right\rangle$ is the ground state of $\mathcal{H}$ and is annihilated by the block matrix $\mathbb{L}_{\mathrm{RE}}$. Hence, we can employ the algorithmic recipe developed in Appendix 2 to prepare the encoded Gibbs state $\left|\sqrt{\boldsymbol{\sigma}}\right\rangle$.

B. Block-encoding of $\mathbb{L}_{\mathrm{RE}}$. In this subsection, we discuss the cost of building a block-encoding of $\mathbb{L}_{\mathrm{RE}}$. The operator matrix $\mathbb{L}_{\mathrm{RE}}$ can be efficiently block-encoded using both the zeroth- and first-order information V. To compute the swapping rate $s(x,y)$, we require access to the function value (i.e., zeroth-order) oracle of V:

$$O_V \colon \ |\boldsymbol{x}\rangle |z\rangle \mapsto |\boldsymbol{x}\rangle |V(\boldsymbol{x})\rangle,$$

and its inverse. Here, $|V(\boldsymbol{x})\rangle$ is a bit-string state that encodes a b-bit representation of $V(\boldsymbol{x})$.

Similar to the Langevin dynamics case, we first perform a spatial discretization of $\mathbb{L}_{\mathrm{RE}}$. Note that each component operator L_j in $\mathbb{L}_{\mathrm{RE}}$ acts on functions in $\mathbb{R}^{2d}$. We first truncate the space $\mathbb{R}^{2d}$ into a finite-sized domain $\Omega = [-a,a]^{2d}$ and discretize it using a uniform grid with N^{2d} points. The discretized L_j operator is a matrix of dimension N^{2d}. For a smooth potential with certain growth conditions, to achieve a target sampling accuracy ϵ in the TV distance, we can choose $a = \mathcal{O}(\log(d/\epsilon))$ and $N = a \cdot \mathrm{poly}\log(d/\epsilon)$ We denote $R = \max_{x\in\Omega} \|\nabla V(x)\|$ as the sub-normalization factor of the gradient ∇V in the numerical domain Ω.

Proposition 19 (Block-encoding of $\mathbb{L}_{\mathrm{RE}}$). *Let*

$$\alpha = \left(\pi^2 N^2 d(\beta^{-1} + \beta'^{-1}) + (\beta+\beta')R^2/4 + 2\pi N\sqrt{d}R + 2\mu\right)^{1/2}, \quad [68]$$

where N and R are the same as above. We can implement an $(\alpha, 10)$-block-encoding of the (discretized) $\mathbb{L}_{\mathrm{RE}}$ using 2 queries to the gradient oracle $O_{\nabla V}$ or its inverse, 4 queries to the function value oracle O_V or its inverse, and an additional $\widetilde{\mathcal{O}}(d^2)$ elementary gates.

Proof. For $j \in [d]$, the block-encoding of L_j and L'_j can be constructed using a similar method as described in Proposition 15. It is worth noting that, while we have two replicas and each of them requires an independent gradient ∇V, they can still be achieved by 2 queries of $P_{\nabla V}$. This is because we can apply two rounds of "controlled" rotation gadgets, each for a replica system, with the same gradient information in the ancilla sub-register (see Eq. Eq. (41)-Eq. (46)). The block-encodings of L_j and L'_j have subnormalization factors

$$\alpha_1 = \pi N\sqrt{d}\beta^{-1/2} + \beta^{1/2}R/2, \quad \alpha_2 = \pi N\sqrt{d}\beta'^{-1/2} + \beta'^{1/2}R/2, \quad [69]$$

respectively, where $R = \max_{x\in\Omega}\|V(x)\| \le \ell\sqrt{d}\log(d)$. Each set of operators $\{L_j\}_{j=1}^d$ and $\{L'_j\}_{j=1}^d$ requires $m_1 = 3$ ancilla qubits. According to Proposition 15, the number of elementary gates is $\tilde{\mathcal{O}}(d^2)$.

Now, we discuss how to block-encode the operator $L_s = \sqrt{\mu/2}(I-W)S^{1/2}$. Recall that S is the multiplication operator given by $[S\psi](x,y) \coloneqq s(x,y)\psi(x,y)$. Note that the function $s(x,y)$ in Eq. (64) can be computed by 2 queries to the function value of V (one for $V(x)$, another for $V(y)$). We can construct an arithmetic circuit that computes

$$|x,y\rangle |0,0,0\rangle \mapsto |x,y\rangle \left|V(x), V(y), \sqrt{s(x,y)}\right\rangle. \quad [70]$$

Since $|s(x,y)| \le 1$, we can introduce an extra ancilla qubit and apply a sequence of rotations to generate an amplitude for the $|0\rangle$ state that equals the value of $\sqrt{s(x,y)}$:

$$|x,y\rangle \left|V(x), V(y), \sqrt{s(x,y)}\right\rangle |0\rangle \mapsto |x,y\rangle \left|V(x), V(y), \sqrt{s(x,y)}\right\rangle \left(\sqrt{s(x,y)}\,|0\rangle + \sqrt{1-s(x,y)}\,|1\rangle\right). \quad [71]$$

Finally, we uncompute the middle register (i.e., $\left|V(x), V(y), \sqrt{s(x,y)}\right\rangle$) by inverting the arithmetic circuit computing $\sqrt{s(x,y)}$ and another 2 uses of the inverse O_V. This realizes a $(1,1)$-block-encoding of the multiplication operator $S^{1/2}$:

$$|x,y\rangle |0\rangle \mapsto \sqrt{s(x,y)}\,|x,y\rangle |0\rangle + |\perp\rangle |1\rangle, \quad [72]$$

with a total number of $\mathrm{poly}(b)$ elementary gates, where b is the fixed-point precision to represent real numbers (e.g., $V(x)$, $\sqrt{s(x,y)}$). The swap operator W can be implemented by $d\log_2(N)$ swap gates without ancilla qubits, where N is the

discretization number and $\log_2(N)$ is the number of qubits representing the quadrature states $|x\rangle$ (or $|y\rangle$). Using the standard Linear Combination of Unitaries (LCU) (44, Lemma 52) , we can implement a $(1,1)$-block-encoding of $I - W$. Overall, we can implement a block-encoding of L_s with 4 queries to O_V, $\mathcal{O}(d\log(N))$ elementary gates, and $m_2 = 2$ ancilla qubits (one for the phase $\sqrt{s(x,y)}$, another for LCU). The subnormalization factor of this block-encoding is

$$\alpha_3 = \sqrt{2\mu}. \quad [73]$$

We can make all elementary gates in the block-encoding of L_s to be controlled on $|2d+1\rangle$, then by concatenating this "select-$(2d{+}1)$" oracle with a basis change that maps $|0\rangle$ to $|2d+1\rangle$, we obtain a unitary that maps $|0^{m_3}\rangle|\psi\rangle$ to $|2d+1\rangle(L_s/\alpha_3)|\psi\rangle + |\perp\rangle$, i.e., it block-encodes L_s (with subnormalization factor α_3) in the $(2d+1)$-th block in the first column.

The last step is to construct the full block-encoding of $\mathbb{L}_{\mathrm{RE}}$ by stacking the above three parts together. We denote the the block-encodings for $\{L_j\}_{j=1}^d$, $\{L_j'\}_{j=1}^d$, and L_s by U_1, U_2, and U_s, respectively. Now, we add 2 ancilla qubits as the control register and form a "select" oracle of the form:

$$\boldsymbol{U} = |00\rangle\langle 00| \otimes U_1 + |01\rangle\langle 01| \otimes U_2 + |10\rangle\langle 10| \otimes U_s + |11\rangle\langle 11| \otimes I. \quad [74]$$

Now, we define a state $|\boldsymbol{\alpha}\rangle = \frac{\alpha_1}{\alpha}|00\rangle + \frac{\alpha_2}{\alpha}|01\rangle + \frac{\alpha_3}{\alpha}|10\rangle$ with a normalization factor $\alpha = \sqrt{\alpha_1^2+\alpha_2^2+\alpha_3^2}$, as given in Eq. (68). Note that $|\boldsymbol{\alpha}\rangle$ is a 2-qubit state and can be prepared by a circuit with $\mathcal{O}(1)$ elementary rotation gates. We denote this state preparation circuit as $\boldsymbol{P}$. By applying the unitary operator $\boldsymbol{U}$ on the state $|\boldsymbol{\alpha}\rangle|0^m\rangle|\psi\rangle$ with $m = 2m_1 + m_2 = 8$, we end up with:

$$\boldsymbol{U}|\boldsymbol{\alpha}\rangle|0^m\rangle|\psi\rangle = |\boldsymbol{\alpha}\rangle|0^m\rangle\left(\frac{1}{\alpha}\sum_{j=1}^{2d+1} A_j|\psi\rangle\right) + |\perp\rangle, \quad A_j = \begin{cases} L_j, & j\in\{1,\dots,d\} \\ L_j', & j\in\{d+1,\dots,2d\} \\ L_s, & j\in\{2d+1\}. \end{cases} \quad [75]$$

Finally, by uncomputing $|\boldsymbol{\alpha}\rangle$ by $\boldsymbol{P}^\dagger$, the circuit $\boldsymbol{P}^\dagger\boldsymbol{U}\boldsymbol{P}$ implements a $(\alpha, 10)$-block-encoding of $\mathbb{L}_{\mathrm{RE}}$. In total, we have used 2 queries to $O_{\nabla V}$, 4 queries to O_V, and an additional $\widetilde{\mathcal{O}}(d^2)$ elementary gates. □

C. Proof of Theorem 2. Similar to the previous section, we make the following assumption to ensure the efficiency of the spatial discretization of the generalized Witten Laplacian of RELD:

Assumption 20 (Spatial discretization of RELD)**.** *Let $V\colon \mathbb{R}^d \to \mathbb{R}$ be a smooth potential function, and $\boldsymbol{\sigma} \propto e^{-\beta V(x) - \beta' V(y)}$ be the joint Gibbs measure. For an arbitrary $\epsilon > 0$, we assume that we can choose $a = \mathcal{O}(\log(d/\epsilon))$ and $N = a\cdot \mathrm{poly}\log(d/\epsilon)$ such that the followings hold:*

1. *Let $\widetilde{\mathbb{L}}_{\mathrm{RE}}$ be discretized $\mathbb{L}_{\mathrm{RE}}$ (as in Eq. (67)) described in Proposition 19. The operator $\widetilde{\mathcal{H}}_{\mathrm{RE}} = \widetilde{\mathbb{L}}_{\mathrm{RE}}^\dagger\widetilde{\mathbb{L}}_{\mathrm{RE}}$ (i.e., discretized Witten Laplacian of RELD) has a ground state $\left|\widetilde{\sqrt{\boldsymbol{\sigma}}}\right\rangle$ that satisfies $\left\| I_N \left|\widetilde{\sqrt{\boldsymbol{\sigma}}}\right\rangle - \sqrt{\boldsymbol{\sigma}}\right\|_{L^2} \le \epsilon/4$,*
2. *Compared to the Witten Laplacian of RELD: $\mathcal{H}_{\mathrm{RE}} = \mathbb{L}_{\mathrm{RE}}^\dagger\mathbb{L}_{\mathrm{RE}}$, the smallest eigenvalue of $\widetilde{\mathcal{H}}_{\mathrm{RE}}$ is no greater than $\mathrm{Gap}(\mathcal{H}_{\mathrm{RE}})/16$, and the second smallest eigenvalue of $\widetilde{\mathcal{H}}$ is no smaller than $9\mathrm{Gap}(\mathcal{H}_{\mathrm{RE}})/16$.*

The following result is a detailed version of Theorem 2.

Theorem 21. *Suppose that Assumption 20 holds and the potential V is ℓ-smooth. Assume access to a state $|\boldsymbol{\phi}\rangle$ (i.e., warm start) such that $\left|\left\langle \boldsymbol{\phi}\middle|\sqrt{\boldsymbol{\sigma}}\right\rangle\right| = \Omega(1)$. Let $\mathcal{L}$ be the generator of RELD as defined in Eq. (57). Then, there exists a quantum algorithm that outputs a random variable $X \in \mathbb{R}^d$ distributed according to η such that $\mathrm{TV}(\eta,\sigma) \le \epsilon$ using*

$$\sqrt{\frac{d}{\mathrm{Gap}(\mathcal{L}^\dagger)}}\cdot\left(1/\beta' + \ell^2\beta + \mu/d\right)^{1/2}\cdot \mathrm{poly}\log\left(d,\epsilon^{-1}\right) \quad [76]$$

quantum queries to the function value V and gradient ∇V, respectively.

Proof. By Proposition 19, we can construct a $(\alpha, 10)$-block-encoding of $\widetilde{\mathbb{L}}_{\mathrm{RE}}$ with a normalization factor

$$\alpha \le 2\pi N\sqrt{d}\beta^{-1/2} + \beta^{1/2}R + \sqrt{2\mu}. \quad [77]$$

Since V is ℓ-smooth and is restricted to the box-shaped numerical domain Ω, we have $R = \max_{x\in\Omega}\|\nabla V(x)\| = \mathcal{O}(\ell\sqrt{d}\log(d))$. It turns out that

$$\alpha = \mathcal{O}\left(N\sqrt{d}\cdot\log(d)\cdot\left(1/\beta' + \ell^2\beta + \mu/d\right)^{1/2}\right). \quad [78]$$

Recall that the right singular vector of $\widetilde{\mathbb{L}}_{\mathrm{RE}}$ associated with the smallest singular value is denoted by $\left|\widetilde{\sqrt{\boldsymbol{\sigma}}}\right\rangle$. Given access to a warm start state $|\boldsymbol{\phi}\rangle$ such that $\left|\left\langle\boldsymbol{\phi}\middle|\sqrt{\boldsymbol{\sigma}}\right\rangle\right| = \Omega(1)$, by Theorem 6, we can prepare a quantum state $|\mathbf{g}\rangle$ that is $\epsilon/4$-close to $\left|\widetilde{\sqrt{\boldsymbol{\sigma}}}\right\rangle$ with

$$\mathcal{O}\left(\frac{\alpha}{\sqrt{\mathrm{Gap}(\mathcal{L}^\dagger)}}\right) = \sqrt{\frac{d}{\mathrm{Gap}(\mathcal{L}^\dagger)}}\cdot\left(1/\beta' + \ell^2\beta + \mu/d\right)^{1/2}\cdot\mathrm{poly}\log\left(\epsilon^{-1}\right)\cdot\log(d) \quad [79]$$

queries to the block-encoding of $\widetilde{\mathbb{L}}_{\mathrm{RE}}$, which amounts to the same query complexity to O_V and $O_{\nabla V}$ due to Proposition 19. By Assumption 20 and the fact that I_N is an isometry, we invoke the triangle inequality to show that

$$\| I_N \left|\mathbf{g}\right\rangle - \sqrt{\boldsymbol{\sigma}} \|_{L^2} \le \epsilon/2. \tag{80}$$

Finally, by Lemma 7, we can realize a random variable $Z = (X, Y) \sim \boldsymbol{\eta}$ such that $\mathrm{TV}(\boldsymbol{\eta}, \boldsymbol{\sigma}) \le \epsilon$ with the number of queries to ∇V and V given by Eq. (76). This immediately implies that the distribution of $X \sim \eta$, given by the marginal law of $\boldsymbol{\eta}$, satisfies $\mathrm{TV}(\eta, \sigma) \le \epsilon$. This concludes the proof. □

5. Details of Lindbladian-Based Warm-Start Preparation

In this section, we establish a direct connection between the Fokker–Planck equation (Eq. [3] in the main text) and the Lindblad master equation (Eq. [17] in the main text). In particular, by representing the density operator as a function in the form $\rho(t, x, y) \coloneqq \langle x|\rho(t)|y\rangle$, we show that the "diagonal element" of this function, namely, $\rho(t, x, x)$, solves the Fokker–Planck equation.

To prove this result, we first introduce some technical notations. Let $w(x)\colon \mathbb{R}^d \to \mathbb{R}$ be a real-valued function, we denote $\hat{w}$ as the corresponding multiplicative operator acting on a test function φ pointwisely, i.e., $(\hat{w}\varphi)(x) = w(x)\varphi(x)$.

Lemma 22. *Let $\rho(t)$ be the solution to the Lindblad master equation (Eq. [17] in the main text), and $p(t, x)$ be the solution to Eq. [3] (main text) with initial condition $p(0, x)$ given by the diagonal of $\rho(0)$. Then, for any smooth function $w(x)\colon \mathbb{R}^d \to \mathbb{R}$ and $t \ge 0$, we have*

$$\mathrm{Tr}[\hat{w}\rho(t)] = \int w(x) p(t, x)\,\mathrm{d}x. \tag{81}$$

Proof. Since $p(0, x)$ matches the distribution given by $\rho(0)$, we have

$$\mathrm{Tr}[\hat{w}\rho(0)] = \int w(x)\rho(0, x, x)\,\mathrm{d}x = \int w(x) p(0, x)\,\mathrm{d}x, \tag{82}$$

which implies that Eq. (81) holds at $t = 0$. Next, we note that

$$\partial_t \,\mathrm{Tr}[\hat{w}\rho(t)] = \mathrm{Tr}\left[\hat{w}\mathfrak{L}[\rho(t)]\right] = \mathrm{Tr}\left[\mathfrak{L}^\dagger[\hat{w}]\rho(t)\right], \tag{83}$$

where $\mathfrak{L}^\dagger$ denotes the adjoint of the Lindbladian operator $\mathfrak{L}$: for any observable O, we have

$$\mathfrak{L}^\dagger[O] = \sum_{j=1}^{d} \left(2L_j^\dagger O L_j - \{L_j^\dagger L_j, O\}\right).$$

Recall that $L_j = -i\frac{1}{\sqrt{\beta}}\partial_{x_j} - i\frac{\sqrt{\beta}}{2}\partial_{x_j} V$, $L_j^\dagger = -i\frac{1}{\sqrt{\beta}}\partial_{x_j} + i\frac{\sqrt{\beta}}{2}\partial_{x_j} V$. For a fixed $j \in [d]$, direct calculation shows that

$$L_j^\dagger \hat{w} L_j - \frac{1}{2}\{L_j^\dagger L_j, \hat{w}\} = -\frac{1}{2}(\partial_{x_j} V)(\partial_{x_j} w) + \frac{1}{2\beta}(\partial_{x_j}^2 w) \tag{84}$$

therefore, we have

$$\mathfrak{L}^\dagger[\hat{w}] = -\nabla V \cdot \nabla w + \frac{1}{\beta}\Delta w. \tag{85}$$

It follows that

$$\partial_t \,\mathrm{Tr}[\hat{w}\rho(t)] = \int \left(-\nabla V \cdot \nabla w + \frac{1}{\beta}\Delta w\right)\rho(t, x, x)\,\mathrm{d}x \tag{86}$$

$$= \int w(x)\left(\nabla \cdot (p(t, x)\nabla V) + \frac{1}{\beta}\Delta p(t, x)\right)\mathrm{d}x, \tag{87}$$

where we write $p(t, x) = \rho(t, x, x)$ and the last step uses integration by parts. Comparing Eq. (86) with the Fokker–Planck equation (Eq. [3] in the main text), we find that $p(t, x)$ solves the Fokker–Planck equation and thus Eq. (81) holds. □

6. Details of Numerical Experiments

A. Numerical simulation of quantum algorithms. In this subsection, we provide some details of the numerical simulation of our quantum algorithms on a classical computer. All experiments were conducted using MATLAB 2024b on a machine equipped with Intel(R) Core(TM) i7-14700K with 64 GB of memory.

Spatial discretization of $\mathbb{L}$. In our experiments, differential operators (e.g., ∂_x) are represented as pseudo-differential operators and computed using Discrete Fourier Transform (DFT). With a regular mesh grid, operators defined as point-wise multiplication (e.g., $\partial_j V$ in LD or $S^{1/2}$ in RELD) are discretized and represented as diagonal matrices. For details, see the discussions in Section A.

Numerical implementation of singular value thresholding. The numerical implementation of the quantum singular value transformation (QSVT) involves approximating the desired operator transformations via polynomial approximations. Specifically, we construct a suitable Chebyshev polynomial summation $P(x)$ to approximate the relevant spectral filtering function by Fourier-Chebyshev expansion method (51, Section III.3): To approximate a smooth real-valued function $F(x)$ defined on the interval $[-1, 1]$, we express it in terms of Chebyshev polynomials of the first kind, i.e.,

$$F(x) \approx P(x) = \sum_{j=0}^{d} c_j T_j(x), \qquad [88]$$

where $T_j(x)$ denotes the Chebyshev polynomial of degree j. The coefficients c_j can be efficiently computed using the fast Fourier transform (FFT) and a quadrature-based formula:

$$c_j \approx \frac{(2-\delta_{j0})}{2K}(-1)^j \sum_{l=0}^{2K-1} F(-\cos\theta_l) e^{ij\theta_l} \qquad [89]$$

Here, the quadrature nodes are given by $\theta_l = \pi l/K$, where l ranges from 0 to $2K-1$, and K represents the total number of quadrature points. In our case, the spectral filtering function is a non-smooth rectangle function given by Eq. (16). Since the Fourier-Chebyshev expansion method applies to smooth functions, we first pre-process the rectangular filter function using cosine-based smoothing near transition points, yielding a smoothened filter function $F(x)$:

$$F(x) = \begin{cases} 0, & x \in [-1, -s_1-\delta] \cup [s_2+\delta, 1] \\ \frac{1}{2}\left[1 - \cos\left(\pi \frac{x-(-s_1-\delta)}{\delta}\right)\right], & x \in [-s_1-\delta, -s_1], \\ 1, & x \in [-s_1, s_2], \\ \frac{1}{2}\left[1 + \cos\left(\pi \frac{x-s_2}{\delta}\right)\right], & x \in [s_2, s_2+\delta]. \end{cases} \qquad [90]$$

This new filter function $F(x)$ and its polynomial approximation $P(x)$ given by Eq. (88) are good approximations to the rectangular filter when δ is small. Meanwhile, the polymomial $P(x)$ satisfies Lemma 4 after appropriately rescaling and shifting $P(x)$ to ensure $|P(x)| \leq 1$.

To numerically implement singular value thresholding, we first compute the singular value decomposition of $\mathbb{L} = W\Sigma V^\dagger$ and apply the polynomial filtering function $P(x)$ to the singular values Σ. The desired (approximate) projector onto the encoded Gibbs state is given by $\tilde{\Pi} = VP(\Sigma)V^\dagger$, as described in Section C. Finally, we apply the operator $\tilde{\Pi}$ to the warm start state $|\phi\rangle$ to obtain the desired state, which is a close approximation of the true Gibbs state $\left|\sqrt{\sigma}\right\rangle$.

Limitations of classical simulation. Despite these numerical strategies, classical simulations of the quantum algorithm remain fundamentally limited by the exponential growth of memory as dimension increases. In our experiments, the size of the component operators in the discretized $\mathbb{L}$ is N^d for LD and N^{2d} for RELD. In both cases, the matrix size grows exponentially with the problem dimension d. The numerical simulation becomes memory-intensive when involving (pseudo)differential operators of dimension $d \geq 3$. Thus, we implement numerical simulations of our quantum algorithms for two test problems: the 2-dimensional Müller-Brown potential (for LD) and a 1-dimensional non-convex potential (for RELD).

B. Details of quantum-accelerated Langevin dynamics. The analytic expression of Müller-Brown potential (52, Footnote 7) is given by

$$V(x,y) = \sum_{k=1}^{4} A_k \exp\left[a_k(x-x_k^0)^2 + b_k(x-x_k^0)(y-y_k^0) + c_k(y-y_k^0)^2\right], \qquad [91]$$

with the constants:

$$\begin{aligned} A &= (-200, -100, -170, 15), & a &= (-1, -1, -6.5, 0.7), \\ b &= (0, 0, 11, 0.6), & c &= (-10, -10, -6.5, 0.7), \\ x^0 &= (1, 0, -0.5, -1), & y^0 &= (0, 0.5, 1.5, 1). \end{aligned}$$

The landscape of the potential is shown in Fig. S2.

In numerical experiments of MALA, we select a time step size $\Delta t = 10^{-3}$ to ensure numerical stability and adequate accuracy in sampling trajectories. The total number of generated samples is set to 3×10^4, sufficiently large to approximate equilibrium distributions accurately. We explore various inverse temperature parameters $\beta \in \{0.4, 0.6, 0.8\}$. The MATLAB `histcounts2` function is utilized here to analyze the sampled data, which partitions the sampled values into $N = 50$ discrete bins and counts the frequency of samples within each bin to get distribution. The frequency is used as an estimation of the sample distribution.

For quantum-accelerated Langevin dynamics, we set the number of grids to $N = 50$ (or 50 Fourier modes) and try different degrees of polynomial to approximate the rectangle function Eq. (16). A subtle but important point is that we found $\mathrm{Gap}(\mathbb{L})/\alpha$

to be too small to be effectively filtered. This is because the normalization factor α becomes very large due to the influence of $R = \max_{\boldsymbol{x}\in\Omega} \|\nabla V(\boldsymbol{x})\|$. As shown in Fig. S2, R is primarily determined by the gradient in the top-right yellow region of the Müller-Brown potential. However, it is unlikely that MALA or quantum-accelerated Langevin dynamics would explore this region, as the potential values there are extremely high. To mitigate the effect of the normalization factor, we slightly modifiy the potential to $\max(V(x), 5)$. This effectively reduces α, which reduces the polynomial degree of the filter function without affecting the quality of the Gibbs state. Then, we follow the methodology described in Section A to implement quantum-accelerated Langevin dynamics. The results are presented in Figure 4 (main text).

C. Details of quantum-accelerated replica exchange. In our numerical implementation of quantum-accelerated RELD, we set the number of grids (or Fourier modes) to be $N = 150$. We use two replicas: one corresponds to a low-temperature chain with inverse temperature β, and another corresponds to a high-temperature chain with inverse temperature β'. We fix the inverse temperature of the high-temperature chain $\beta' = 1$ and vary β for the low-temperature chain from 2 to 10. The swapping intensity is also fixed as $\mu = 1$. To compute the spectral gaps, we perform singular value decomposition on the operator $\mathcal{H}$ (see Eq. [14] in the main text) for classical RELD, and on $\mathbb{L}_{\mathrm{RE}}$ (see Eq. [16] in the main text) for quantum-accelerated RELD.

D. Details of Lindbladian-based warm-start preparation. This part numerically solves the Lindblad equation (see Eq. [17] in the main text) when the potential function is Eq. [18] in the main text. We adopt the spatial discretization method described in Section A with the number of grids (or Fourier modes) $N = 50$. The time step size is $\Delta t = 1 \times 10^{-4}$, and the time propagation is conducted using the 4th-order Runge-Kutta method. The initial distribution is taken to be a very sharp Gaussian centered at $x = -1.7$, whose initial overlap with the Gibbs distribution is smaller than 3×10^{-2}. Here, we set the inverse temperature β from 2 to 10, which covers the range of the numerical experiment shown in Figure 5b. The overlap is calculated using Eq. [19] (main text), and the numerical values are shown in Figure 6b.

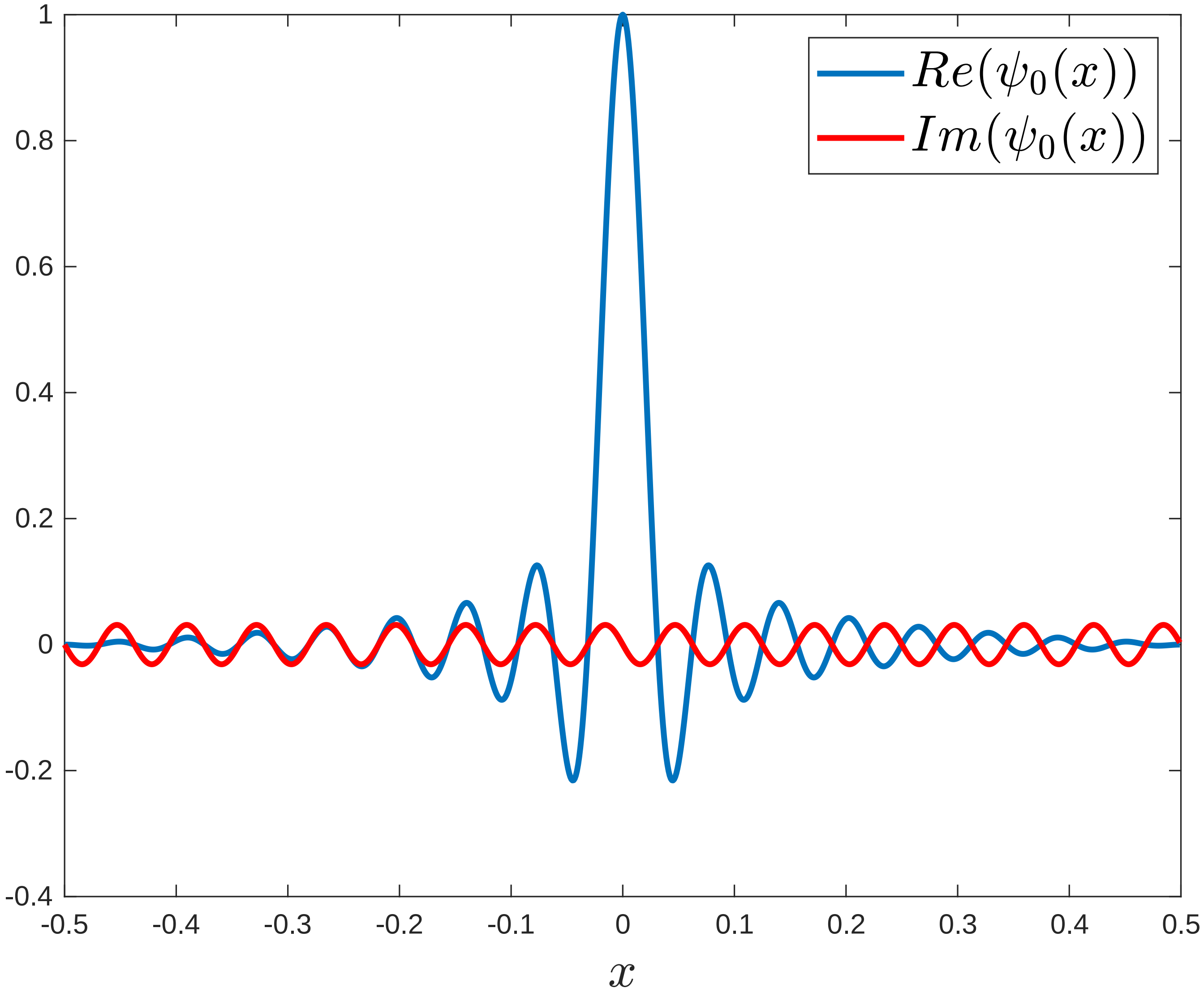

Fig. S1. Real and imaginary parts of the trigonometric polynomial $\psi_0(x)$ (normalized such that $\psi_0(0) = 1$) over $[-1/2, 1/2]$ with $N = 32$.

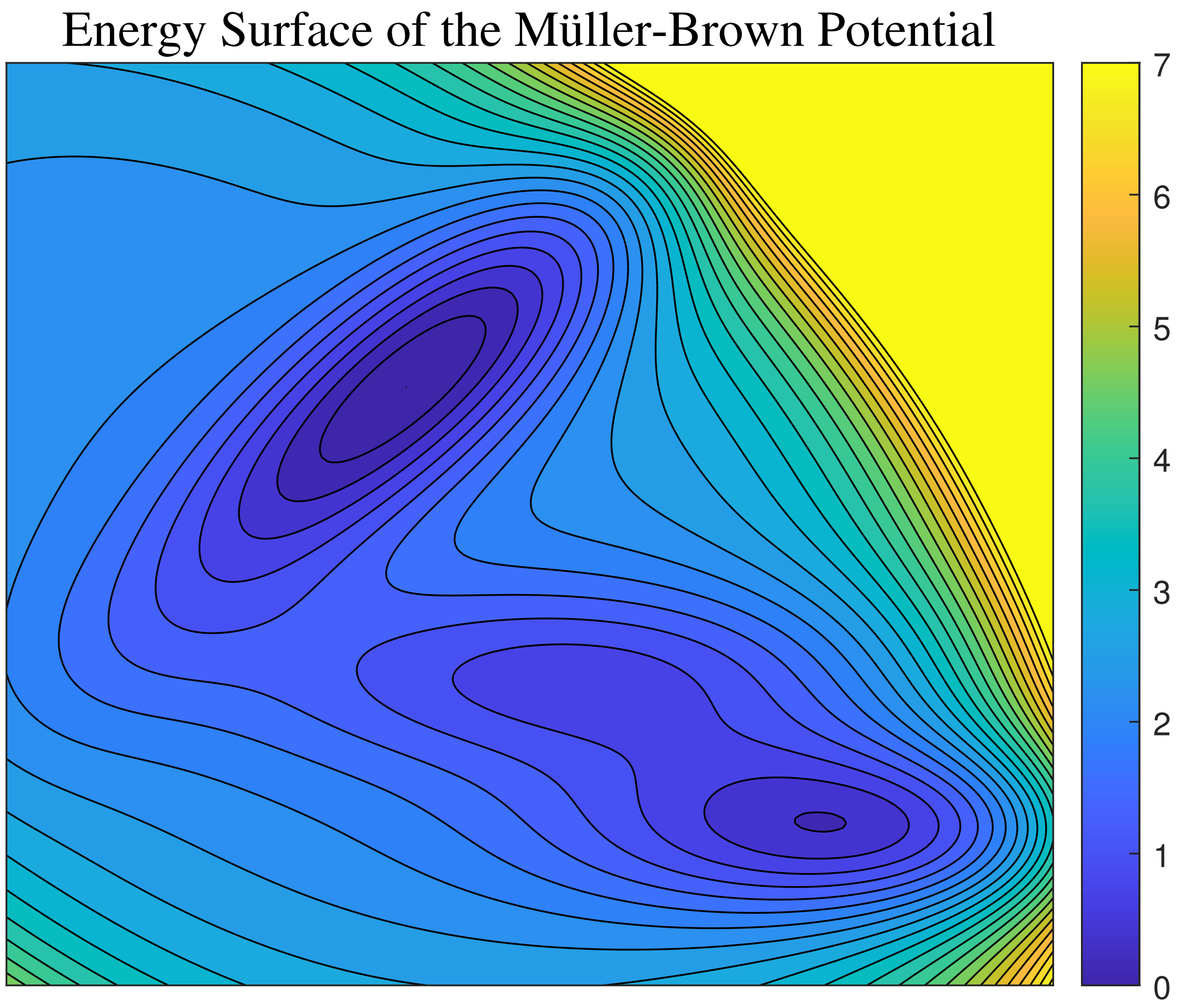

Fig. S2. Energy surface of Müller-Brown potential (with truncated color scale).

References

1. L Breiman, *Probability.* (Society for Industrial and Applied Mathematics), (1992).
2. P Billingsley, *Convergence of probability measures*, Wiley Series in Probability and Statistics: Probability and Statistics. (John Wiley & Sons Inc., New York), pp. x+277 (1999) A Wiley-Interscience Publication.
3. LN Vaserstein, Markov processes over denumerable products of spaces, describing large systems of automata. *Probl. Peredachi Informatsii* **5**, 64–72 (1969).
4. LV Kantorovich, Mathematical methods of organizing and planning production. *Manag. Sci.* **6**, 366–422 (1960).
5. T Morimoto, Markov processes and the h-theorem. *J. Phys. Soc. Jpn.* **18**, 328–331 (1963).
6. A Rényi, On measures of entropy and information in *Proceedings of the Fourth Berkeley Symposium on Mathematical Statistics and Probability.* (University of California Press), Vol. 1, pp. 547–561 (1961).
7. SM Ali, SD Silvey, A general class of coefficients of divergence of one distribution from another. *J. Royal Stat. Soc. Ser. B (Methodological)* **28**, 131–142 (1966).
8. I Csiszar, I-Divergence geometry of probability distributions and minimization problems. *The Annals Probab.* **3**, 146 – 158 (1975).
9. NG van Kampen, *Stochastic Processes in Physics and Chemistry.* (North-Holland, Amsterdam), 2nd edition, (1992).
10. A O'Hagan, JJ Forster, *Kendall's Advanced Theory of Statistics, Volume 2B: Bayesian Inference.* (Arnold, London), 2nd edition, (2004).
11. C Villani, *Hypocoercivity.* (American Mathematical Society) Vol. 202, (2009).
12. C Villani, *Optimal Transport: Old and New*, Grundlehren der mathematischen Wissenschaften. (Springer, Berlin, Heidelberg) Vol. 338, (2009).
13. M Ledoux, *The Concentration of Measure Phenomenon*, Mathematical Surveys and Monographs. (American Mathematical Society, Providence, RI) Vol. 89, (2001).
14. D Bakry, I Gentil, M Ledoux, *Analysis and Geometry of Markov Diffusion Operators*, Grundlehren der mathematischen Wissenschaften. (Springer, Cham) Vol. 348, (2014).
15. S Chewi, Log-concave sampling. *Book draft available at https://chewisinho. github. io* (2023).
16. AM Childs, T Li, JP Liu, C Wang, R Zhang, Quantum algorithms for sampling log-concave distributions and estimating normalizing constants. *Adv. Neural Inf. Process. Syst.* **35**, 23205–23217 (2022).
17. G Ozgul, X Li, M Mahdavi, C Wang, Stochastic quantum sampling for non-logconcave distributions and estimating partition functions in *Proceedings of the 41st International Conference on Machine Learning*, Proceedings of Machine Learning Research, eds. R Salakhutdinov, et al. (PMLR), Vol. 235, pp. 38953–38982 (2024).
18. G Ozgul, X Li, M Mahdavi, C Wang, Quantum speedups for Markov chain Monte Carlo methods with application to optimization. *arXiv/2504.03626* (2025).
19. J Cheeger, A lower bound for the smallest eigenvalue of the Laplacian in *Problems in Analysis (Papers Dedicated to Salomon Bochner)*, ed. RC Gunning. (Princeton University Press, Princeton, NJ), pp. 195–199 (1969).
20. DA Levin, Y Peres, EL Wilmer, *Markov Chains and Mixing Times*, American Mathematical Society Textbooks. (American Mathematical Society, Providence, RI), Second edition, (2017).
21. M Ledoux, Spectral gap, logarithmic Sobolev constant, and geometric bounds in *Surveys in Differential Geometry.* (International Press) Vol. 9, pp. 219–240 (2004).
22. E Milman, On the role of convexity in isoperimetry, spectral gap and concentration. *Invent. mathematicae* **177**, 1–43 (2009).
23. R Jordan, D Kinderlehrer, F Otto, The variational formulation of the Fokker–Planck equation. *SIAM J. on Math. Analysis* **29**, 1–17 (1998).
24. A Wibisono, Sampling as optimization in the space of measures: The Langevin dynamics as a composite optimization problem in *Annual Conference Computational Learning Theory.* (2018).
25. YA Ma, et al., Is there an analog of Nesterov acceleration for gradient-based MCMC? *Bernoulli* **27**, 1942 – 1992 (2021).
26. R Shen, YT Lee, The randomized midpoint method for log-concave sampling in *Advances in Neural Information Processing Systems*, eds. H Wallach, et al. (Curran Associates, Inc.), Vol. 32, (2019).
27. Y Cao, J Lu, L Wang, Complexity of randomized algorithms for underdamped Langevin dynamics. *Commun. Math. Sci.* **19**, 1827–1853 (2021).
28. S Chewi, MA Erdogdu, M Li, R Shen, MS Zhang, Analysis of Langevin Monte Carlo from Poincaré to log-Sobolev. *Foundations Comput. Math.* pp. 1–51 (2024).
29. J Besag, P Green, D Higdon, K Mengersen, Bayesian Computation and Stochastic Systems. *Stat. Sci.* **10**, 3 – 41 (1995).
30. GO Roberts, JS Rosenthal, Optimal scaling of discrete approximations to Langevin diffusions. *J. Royal Stat. Soc. Ser. B: Stat. Methodol.* **60**, 255–268 (2002).
31. R Dwivedi, Y Chen, MJ Wainwright, B Yu, Log-concave sampling: Metropolis-Hastings algorithms are fast! in *Proceedings of the 31st Conference On Learning Theory*, Proceedings of Machine Learning Research, eds. S Bubeck, V Perchet, P Rigollet. (PMLR), Vol. 75, pp. 793–797 (2018).
32. Y Chen, R Dwivedi, MJ Wainwright, B Yu, Fast mixing of Metropolized Hamiltonian Monte Carlo: benefits of multi-step gradients. *J. Mach. Learn. Res.* **21** (2020).
33. S Chewi, et al., Optimal dimension dependence of the Metropolis-Adjusted Langevin Algorithm in *Annual Conference Computational Learning Theory.* (2020).

34. YT Lee, R Shen, K Tian, Logsmooth gradient concentration and tighter runtimes for Metropolized Hamiltonian Monte Carlo in *Proceedings of Thirty Third Conference on Learning Theory*, Proceedings of Machine Learning Research, eds. J Abernethy, S Agarwal. (PMLR), Vol. 125, pp. 2565–2597 (2020).
35. K Wu, S Schmidler, Y Chen, Minimax mixing time of the Metropolis-Adjusted Langevin algorithm for log-concave sampling. *J. Mach. Learn. Res.* **23** (2022).
36. Y Chen, K Gatmiry, When does Metropolized Hamiltonian Monte Carlo provably outperform Metropolis-adjusted Langevin algorithm? (2023).
37. JM Altschuler, S Chewi, Faster high-accuracy log-concave sampling via algorithmic warm starts. *J. ACM* **71** (2024).
38. MK Titsias, O Papaspiliopoulos, Auxiliary gradient-based sampling algorithms. *J. Royal Stat. Soc. Ser. B: Stat. Methodol.* **80**, 749–767 (2018).
39. YT Lee, R Shen, K Tian, Structured logconcave sampling with a restricted Gaussian oracle in *Proceedings of Thirty Fourth Conference on Learning Theory*, Proceedings of Machine Learning Research, eds. M Belkin, S Kpotufe. (PMLR), Vol. 134, pp. 2993–3050 (2021).
40. Y Chen, S Chewi, A Salim, A Wibisono, Improved analysis for a proximal algorithm for sampling in *Proceedings of Thirty Fifth Conference on Learning Theory*, Proceedings of Machine Learning Research, eds. PL Loh, M Raginsky. (PMLR), Vol. 178, pp. 2984–3014 (2022).
41. D Zou, P Xu, Q Gu, Faster convergence of stochastic gradient Langevin dynamics for non-log-concave sampling. *Uncertain. artificial intelligence* (year?).
42. L Pillaud-Vivien, F Bach, T Lelièvre, A Rudi, G Stoltz, Statistical estimation of the Poincaré constant and application to sampling multimodal distributions in *Proceedings of the Twenty Third International Conference on Artificial Intelligence and Statistics.* Vol. 108, pp. 2753–2763 (2020).
43. J Dong, XT Tong, Spectral gap of replica exchange Langevin diffusion on mixture distributions. *Stoch. Process. their Appl.* **151**, 451–489 (2022).
44. A Gilyén, Y Su, GH Low, N Wiebe, Quantum singular value transformation and beyond: exponential improvements for quantum matrix arithmetics in *Proceedings of the 51st Annual ACM SIGACT Symposium on Theory of Computing.* pp. 193–204 (2019).
45. E Tang, K Tian, A CS guide to the quantum singular value transformation in *2024 Symposium on Simplicity in Algorithms (SOSA).* (SIAM), pp. 121–143 (2024).
46. A Eremenko, P Yuditskii, Uniform approximation of sgn(x) by polynomials and entire functions. *arXiv preprint math/0604324* (2006).
47. Y Dong, L Lin, Y Tong, Ground-state preparation and energy estimation on early fault-tolerant quantum computers via quantum eigenvalue transformation of unitary matrices. *PRX Quantum* **3**, 040305 (2022).
48. D Coppersmith, An approximate Fourier transform useful in quantum factoring. *arXiv preprint quant-ph/0201067* (2002).
49. A Motamedi, P Ronagh, Gibbs sampling of continuous potentials on a quantum computer in *Proceedings of the 41st International Conference on Machine Learning*, Proceedings of Machine Learning Research, eds. R Salakhutdinov, et al. (PMLR), Vol. 235, pp. 36322–36371 (2024).
50. MA Nielsen, IL Chuang, *Quantum computation and quantum information.* (Cambridge university press), (2010).
51. Y Dong, X Meng, KB Whaley, L Lin, Efficient phase-factor evaluation in quantum signal processing. *Phys. Rev. A* **103**, 042419 (2021).
52. K Müller, LD Brown, Location of saddle points and minimum energy paths by a constrained simplex optimization procedure. *Theor. chimica acta* **53**, 75–93 (1979).
